# Genome-wide diversity and *Pinna rudis* admixture reveal Thau lagoon as a conservation reservoir for the collapsing fan mussel *Pinna nobilis*

**DOI:** 10.3389/fgene.2026.1970183

**Published:** 2026-10-26

**Authors:** Mathieu Foulquié, Robert Bunet, Maite Vázquez Luis, Ioannis A. Giantsis, Fabio Scarpa, Daria Sanna, Elvira Alvarez Perez, Marco Casu, Jean-Marc Prévot, Nardo Vicente, Stéphane Coupé

**Affiliations:** 1 CNRS, IRD, MIO, Université de Toulon, Aix Marseille University, Marseille, France; 2 Institut Océanographique Paul Ricard, Six-Fours-les-Plages, Var, France; 3 Instituto Español de Oceanografía (IEO, CSIC), Centro Oceanográfico de Baleares, Palma de Mallorca, Spain; 4 Laboratory of Ichthyology and Fisheries, Faculty of Agriculture, Forestry and Natural Environment, Aristotle University of Thessaloniki, Thessaloniki, Greece; 5 Department of Biomedical Sciences, University of Sassari, Sassari, Italy; 6 Department of Innovation, University of Sassari, Olbia, Italy; 7 Département Informatique, Université de Toulon, Toulon, Var, France; 8 Institut Méditerranéen de Biodiversité et Ecologie Marine et Continentale (IMBE), CNRS, IRD, Aix-Marseille Université, Avignon Université, Avignon, France

**Keywords:** admixture, bivalve, critically endangered species, fan mussel, local adaptation, mass mortality event, Pinna

## Abstract

The conservation of *Pinna nobilis* increasingly depends on the Thau lagoon population, which has remained demographically stable and largely escaped the mass mortality events (MMEs) that have devastated open-sea populations across the Mediterranean. Here we assess the genetic diversity in the Thau and Diane lagoons (France), and characterize the genome-wide admixture with *Pinna rudis*. Mitochondrial diversity in Thau, especially at protein-coding genes, closely mirrors that previously documented in open-sea populations, while comparisons of historical and contemporary samples reveal differences in 16S rRNA haplotype composition that are consistent with recent erosion of mitochondrial diversity. Extended mitochondrial and nuclear SNP data confirm and refine earlier evidence of successive historical population expansions. We also report the first evidence of mitochondrial heteroplasmy in *P. nobilis*, identified in an individual carrying both *P. nobilis* and *P. rudis* mitogenomes. In contrast, all five confirmed F1 hybrids retained only *P. nobilis* mitogenomes, indicating asymmetric mitochondrial inheritance during hybridization. Nuclear genomic analyses revealed low but widespread admixture with *P. rudis*, consistent with a history of limited yet persistent gene flow. Candidate introgressed regions were enriched for genes involved in innate immune recognition and signalling, apoptosis, cell adhesion, extracellular matrix organization, and proteostasis, identifying pathways potentially relevant to host resilience that warrant functional investigation. However, the absence of complete disease resistance among admixed individuals indicates that any potential contribution of these regions to disease resilience is likely to be modest and context dependent. These results show that *P. nobilis* retains substantial evolutionary potential despite its recent demographic collapse, and that the standing genetic diversity and admixture preserved in the Thau lagoon could constitute valuable resources for conservation. Future studies linking genomic variation with individual infection outcomes will be essential to determine whether the identified candidate variants directly contribute to disease tolerance and resistance.

## 1 Introduction

The Mediterranean endemic fan mussel *Pinna nobilis* is among the most emblematic species of the Mediterranean Sea, living more than 45 years (Rouanet et al., 2015) and reaching up to 1.2 m in length (Zavodnik et al., 1991). It typically inhabits soft-bottom habitats such as *Posidonia oceanica* meadows, anchoring vertically in the sediment. As a filter feeder capable of processing up to 2,500 L of water per day (Hernandis Caballero, 2021), it improves water quality (Trigos et al., 2014) and supports local biodiversity by providing habitat for associated species (Cos et al., 2008; Rabaoui et al., 2009; Pensa et al., 2022). Larvae disperse in the water column for up to 20 days before settling (Wesselmann et al., 2018), and genetic and oceanographic evidence indicates connectivity among distant western Mediterranean populations (Wesselmann et al., 2018; Kersting et al., 2020).

Although *P. nobilis* remained abundant across much of its range for most of its history (Sanna et al., 2023), it has virtually disappeared from many open-sea areas over the past decade. Mass mortality events (MMEs) have varied in their geographic extent, timing, and associated pathogens, with the first major outbreaks reported in the western Mediterranean and subsequently spreading across much of the species’ range (Künili et al., 2021; Scarpa et al., 2020; Carella et al., 2023a). Mortality has been particularly severe in open-sea populations, where local abundances have declined dramatically, whereas populations inhabiting some lagoons and semi-enclosed environments have shown greater persistence, in some cases reaching high densities (Foulquié et al., 2024; Foulquié et al., 2026; Karadurmuş et al., 2025). The mechanisms underlying this spatial heterogeneity remain unresolved and may reflect differences in pathogen exposure, environmental conditions, potentially interacting with host density and intrinsic variation in individual or population susceptibility (Cabanellas-Reboredo et al., 2019; Coupé et al., 2023; Martínez-Martí et al., 2024). Recurrent MMEs are nevertheless expected to have profound genetic consequences, including the loss of allelic and haplotypic diversity, stochastic changes in the frequencies of rare variants, shifts in the spatial distribution of genetic diversity, and reductions in effective population size (Allendorf et al., 2024; Amos and Balmford, 2001). Conversely, populations that have persisted through successive MMEs may retain substantial genetic diversity despite severe demographic impacts (Peyran et al., 2021; Nebot-Colomer et al., 2025), and may harbour genetic variants potentially associated with enhanced survival or delayed disease progression. Such contrasting demographic and genetic trajectories make persistent populations, such as that inhabiting Thau lagoon, particularly valuable for investigating how demographic history, genetic diversity, and interpopulation gene flow may have contributed to persistence under recurrent disease pressure.

Conserving the species therefore matters not only for its preservation as a threatened Mediterranean bivalve and its associated ecological functions, but also because *P. nobilis* can be considered as a relevant model for understanding the broader consequences of emerging infectious diseases in marine ecosystems. Indeed, its dramatic population collapse illustrates how pathogen emergence (Vetri et al., 2026; Carella et al., 2023b; Andree et al., 2020), potentially in interaction with environmental stressors and other infectious agents (Carella et al., 2023a; Carella et al., 2026), can rapidly reshape the distribution and persistence of marine populations. Insights into the mechanisms of disease susceptibility, pathogen transmission and persistence in remnant populations may consequently inform the conservation of other bivalves increasingly threatened by emerging and invasive pathogens (Vetri et al., 2026) and help identify factors that promote population persistence and recovery following disease outbreaks.

Mitochondrial *cox1*-based studies identified two main clades in *P. nobilis*: clade B, the older and dominant lineage with early Pleistocene diversification, and the more recently diverged, smaller clade A (Azzena et al., 2025). Differences in the frequency and representation of clade A lineages between historical (i.e., before MMEs) and contemporary samples have been reported following recent MMEs, raising the possibility that differential survival among mitochondrial lineages may contribute to observed changes in mitochondrial diversity. Recent studies have also revealed basin-specific historical expansions (Sanna et al., 2023), but more recent demographic fluctuations preceding the MMEs remain poorly resolved, a gap that requires high-resolution analysis of the species’ last remaining populations.

Prior to the collapse of *P. nobilis* in open waters, genetic studies collectively depicted a contrasted picture of population structure across the Mediterranean basin. At the basin scales, the species appears homogeneous across the western Mediterranean and Ionian sea (Wesselmann et al., 2018; Peyran et al., 2021; Sanna et al., 2013), the Adriatic sea, and the Aegean sea and Tunisian coastal waters (Sanna et al., 2023; Sanna et al., 2013), including lagoons (Peyran et al., 2021). Despite this high dispersal capacity and apparent connectivity, analyses based on mitochondrial and nuclear markers have also identified fine-scale genetic structuring in both the western and eastern basins. These patterns suggest a potential influence of environmental constraints, such as hydrodynamic processes and complex coastal topography on population dynamics, variability in recruitment success, and potentially cohesive larval dispersal, as already documented in other highly dispersive marine species (Aglieri et al., 2014; Couvray and Coupé, 2018).

Field monitoring has shown that some rare *P. nobilis* individuals survived few MMEs or succumbed only long after disease onset (Coupé et al., 2023). These observation suggest substantial variation in susceptibility and disease progression among individuals, although the genetic basis of this variation remains poorly understood. Such phenotypic variation has been hypothesized to involve admixture with *P. rudis*, a congeneric species that appears, to date, largely unaffected by MMEs, as do first-generation hybrids (Vázquez-Luis et al., 2021; Coupé et al., 2023; Catan et al., 2024). The occurrence of hybridization suggests that some morphologically identified *P. nobilis* individuals may retain *P. rudis* genomic regions through historical hybridization and subsequent backcrossing. These regions may contribute to variation in host response infection, but whether introgression enhances MMEs resistance remains to be established.

The Thau Lagoon population (Figure 1) is now one of the last natural *P. nobilis* reservoirs and a critical resource for conservation. The lagoon comprises two hydrodynamic sectors: the relatively homogeneous western Grand Etang and the more heterogeneous eastern Eaux Blanches (EB) sector, which connects directly to the Mediterranean via the Sète canal network and experiences the strongest marine influence. Water residence time is shorter in EB (approx. 45 days) than in Grand Etang (approx. 110 days) (Fiandrino et al., 2012), and exchange between sectors is wind-driven and asymmetric, mainly enhanced by north westerly winds (Millet, 1989; Lazure, 1992) and more limited under southeasterly conditions, though finer-scale hydrodynamic subdivisions likely exist beyond this simplified two-sector model (Fiandrino et al., 2012; Guelorget et al., 1994; Audouin, 1962).

**FIGURE 1:**
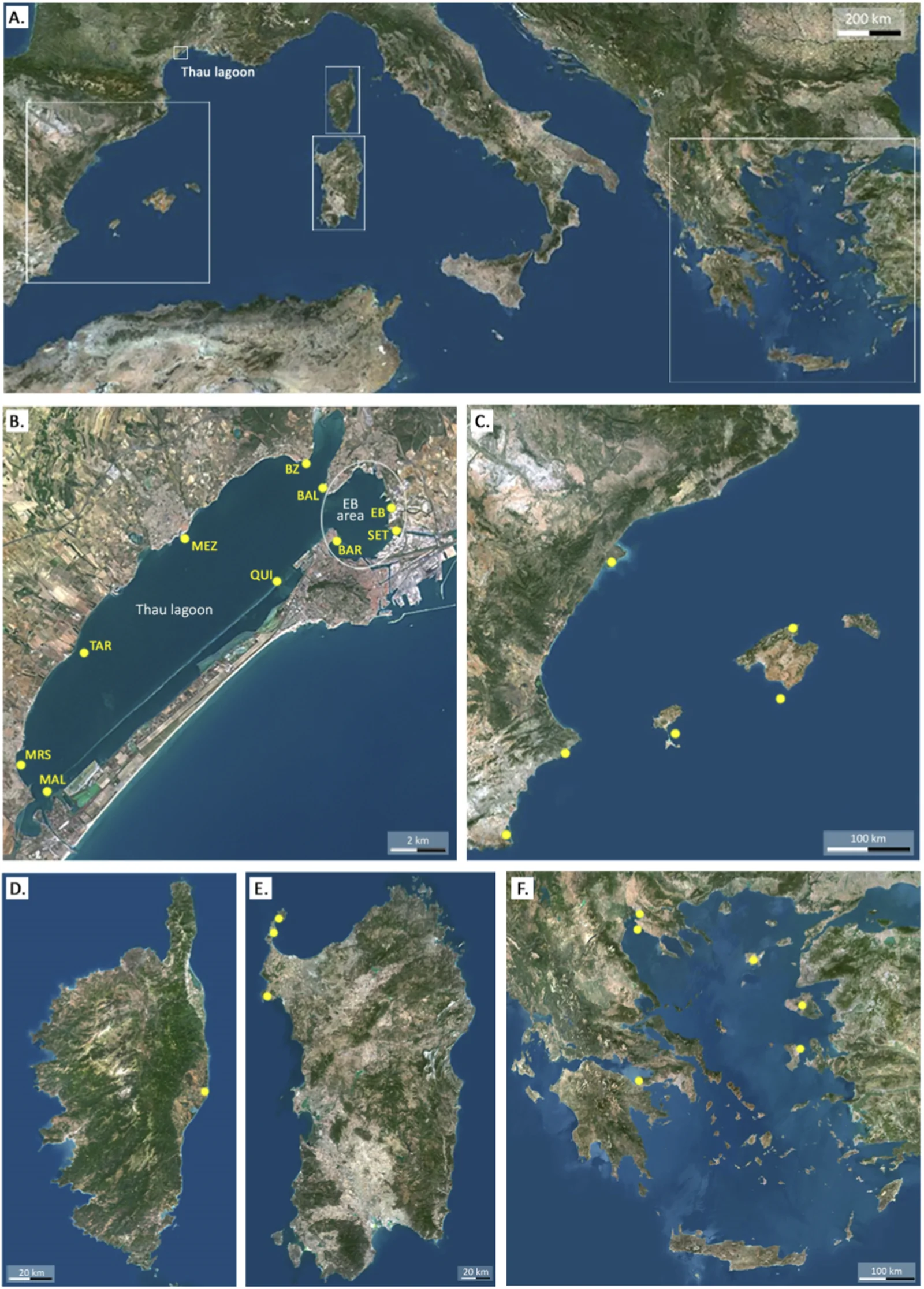
Sampling sites. Overview map showing the geographic distribution of sampling areas across the Mediterranean Sea **(A)**, with detailed maps of the sampling sites in France (Thau and Diane lagoons, **(B)** and **(D)**), respectively), Spain **(C)**, Italy (Sardinia, panel **(E)**), and Greece **(F)**. Yellow dots indicate the locations where mantle tissue biopsies were collected. The white ellipse in the Thau lagoon map indicates the approximate extent of the Eaux Blanches (EB) area.

Recent high-quality genome assemblies for *P. nobilis* and *P. rudis* provide reference genomes that enable genome-wide characterization of genetic diversity and the detection of interspecific admixture and introgression (Coupé et al., 2026). Here, we characterize genome-wide diversity in the Thau lagoon population, examining both maternally and biparentally inherited variation, and compare it with previously reported open-sea diversity to assess whether pre-MME genetic diversity has been retained. We quantify the genomic admixture within *P. nobilis* and identify plausible biological processes associated with introgressed genomic regions. Further, we reconstruct the Thau lagoon population’s demographic history using extended mitochondrial sequences and genome-wide SNPs. We discuss these findings in the context of ongoing efforts to restore *P. nobilis* populations in the open sea.

## 2 Materials and methods

### 2.1 *Pinna* sp. DNA samples, PE-reads resequencing and variant calling

Samplings of *Pinna nobilis*, *Pinna rudis* and *Pinna nobilis* × *Pinna rudis* hybrids were authorized by the relevant French and Spanish authorities. In France, authorization was granted by the Direction régionale de l’environnement, de l’aménagement et du logement d’Occitanie (Arrêté départemental n° 2023-S-8). In Spain, sampling permits included ESP 15/2023 for *Pinna* spp. in the Balearic Islands (2023) and SEN 376/23, issued by Cabrera National Park for sampling conducted within the park in 2023.

Mantle tissue was collected from 334 adult Pinna specimens inhabiting the Thau (n = 301) and Diane (n = 33) lagoons between 2021 and 2025. Within the Thau lagoon, individuals were sampled across 10 sites distributed along the lagoon shoreline (Figure 1B). Mantle tissue was collected *in situ* by freediving or scuba diving using a non-lethal biopsy procedure adapted from previously published protocols for *P. nobilis* (Peyran et al., 2021; Sanna et al., 2013; Maresca et al., 2025; Mihaljević et al., 2021). Individuals were sampled without being dislodged or otherwise manipulated from the substrate. After gently approaching each individual, a small piece of mantle tissue (approximately 6 mm^3^; 15 mg) was rapidly excised from the exposed mantle margin using sterile biopsy forceps before valve closure. Genomic DNA was extracted using the DNAeasy Tissue kit-96 (Qiagen) following manufacturer instruction. DNA integrities were assessed on a fraction of the samples by 0.5% agarose gel electrophoresis. DNA concentrations were measured and qualities were assessed using Nanodrop-1000. Concentrations were normalized to 20 ng/μL. Illumina paired end reads (2 × 150 bp) were generated by Genewiz targeting an expected read depth of 10X. Forty-five previously generated raw sequencing reads from *P. nobilis*, *P. rudis* and F1 hybrid individuals sampled in open-sea habitats from Spain, Italy and Greece, were also incorporated into the dataset (Coupé et al., 2023). In total, 379 individuals were included in this study, comprising 301 from Thau, 33 from Diane, and 45 from open-sea sites (Table 1). These reads were processed and aligned as previously described (Coupé et al., 2023) to the haploid *P. nobilis*-specific component (haplotype 1) of the hybrid reference genome, available through the NCBI BioProject database under accession number PRJNA1527659.

**TABLE 1:** Summary of *Pinna nobilis*, *P. rudis*, and first-generation hybrids (*P. nobilis* × *P. rudis*, F1) samples used in this study. DNA was sequenced using Illumina paired-end sequencing (2 × 150 bp) to achieve at least 10× genome coverage.

Site	Number of individuals sequenced	P. rudis and F1 hybrid	Sequencing year
France (Thau lagoon)	301	​	2021 and 2025
France (Diane lagoon)	33	1 F1 hybrid	2025
Spain (open-sea)	18	2P. rudis, 4 F1 hybrids	2021 and 2025
Greece (open-sea)	13	​	2021
Italy (open-sea)	14	​	2021

Raw single-nucleotide polymorphism (SNP) data were obtained in Variant Call Format (VCF). This raw VCF file was partitioned into a *P. nobilis*-only VCF and a second VCF containing the *P. rudis* (n = 2) and F1 hybrid (n = 5) individuals. This partitioning prevented the marked imbalance in sample size and species composition from influencing variant filtering, particularly for filters based on allele frequencies and missing genotype proportions. For each dataset, only biallelic SNPs were retained. Genotype calls were set to missing if they did not satisfy the following criteria: sequencing depth (DP) between 5 and 60, genotype quality (GQ) ≥ 30, and variant quality score (QUAL) ≥ 30. These thresholds were established based on summary statistics generated with BCFtools (Danecek et al., 2021) stats and were applied to reduce low-confidence genotype calls and sequencing artefacts. Following genotype-level filtering, variants with more than 10% missing genotypes across individuals were removed to minimize bias associated with incomplete genotype information. Minimum allele count (MAC) thresholds ≥5 and >1 were applied to the *P. nobilis*-only and *P. rudis*–F1 hybrid VCF datasets, respectively, to exclude extremely rare variants while retaining low-frequency variants, particularly in the smaller *P. rudis*–F1 hybrid dataset. The two filtered VCF datasets were subsequently merged into a single dataset.

Depending on the downstream analysis, either the merged dataset or the filtered *P. nobilis*-only dataset was used as the starting dataset. Additional analysis-specific filtering, marker thinning, and linkage disequilibrium (LD) pruning were subsequently applied according to the requirements of each method. For analyses of genetic diversity and population structure, a putatively neutral SNP dataset was generated by excluding candidate loci identified as potentially under selection using pcadapt (Luu et al., 2017), whereas the full quality-filtered dataset was retained for ADMIXTURE (Alexander et al., 2009) and RFMix (Maples et al., 2013). The phased and thinned *P. nobilis*-only dataset was used for SMC++ and genomic scans for local adaptation and recent positive selection (Supplementary Figure S1). Detailed filtering and marker-processing steps for each analysis are described in the corresponding sections below.

### 2.2 Mitochondrial diversity analysis

Mitogenome reads were aligned using bwa-mem2 (Li and Durbin, 2009) to the protein-coding (PCG) and ribosomal RNA (rRNA) genes of the *P. nobilis* and *P. rudis* mitogenomes (NCBI accessions PZ220846 and PZ220845, respectively). For each individual and gene, mitochondrial haplotypes were inferred from indexed BAM files using a custom Python pipeline. Gene coordinates were defined in a BED file, overlapping reads were extracted using pysam v0.24.0 and assembled into continuous sequences, with paired end reads merged when possible and read orientations corrected as needed to reconstruct continuous sequences. For each gene within each sample, up to three of the most frequent haplotypes were retained as candidate haplotypes, with corresponding counts and frequencies calculated. This upper limit was used as a conservative measure to account for samples potentially exhibiting complex heteroplasmy. Haplotypes with a frequency higher or equal to 0.1 were subsequently exported as FASTA records for downstream validation by local BLASTn against reference mitogenomes of *P. nobilis* and *P. rudis*. Finally, a combined tab-separated table summarizing haplotype identity, abundance, and frequency across all samples was generated to facilitate the detection of heteroplasmy. Putative heteroplasmy was further confirmed by read-level inspection using the Integrative Genome Viewer (IGV) software (Thorvaldsdóttir et al., 2013).

Then, the *P. nobilis* haplotype of each gene (or concatenated PCG or *rRNA* genes) were aligned with MAFFT v7.471 (--auto, --reorder) via Python v3.13 subprocess, and the resulting alignment was trimmed to retain a conserved core region by excluding terminal columns with <90% site coverage. The trimmed alignment was exported to FASTA and converted to NEXUS format using Biopython v1.85. Haplotypes were defined as unique full-length sequences, and their frequencies were estimated using exact string matching. Additional genetic diversity metrics were calculated using DnaSP v6.10 (Rozas et al., 2017), including haplotype diversity (Hd), nucleotide diversity (Pi), the average number of differences (k), the number of segragating sites (S), and the number of synonymous and non-synonymous substitutions (dS; dN). Haplotype networks were constructed using PopART (Leigh et al., 2015). Similar analyses were performed to generate haplotype networks including the majority of available *cox1* and *16S rRNA* sequences retrieved from the NCBI nucleotide database (April 2026).

### 2.3 Ancient demographic inferences using mitochondrial and nuclear markers

#### 2.3.1 Mitochondrial demographic inference

To infer historical changes in effective population size from mitochondrial data, we estimated Tajima’s *D*, Fu and Li’s *D*, θ, and τ using DnaSP v6.10. These metrics were calculated for both the *16S rRNA* gene and a concatenated dataset of three protein-coding gene (PCG) dataset comprising *nad5*, *cytb*, and *cox1*. Historical demography was further investigated through mismatch distribution analyses and Bayesian phylogenetic inference. Mismatch distributions were generated in DnaSP v6 under a sudden demographic expansion model, for *16S rRNA* gene and the PCG dataset. The fit between the observed and expected distributions was assessed using Harpending’s raggedness index (r), with statistical significance evaluated by coalescent simulations (10,000 replicates) under a no-recombination model with fixed θ. Ninety-five percent confidence intervals were calculated to assess deviations from the expected distribution. Given the limited occurrence of heteroplasmy and the absence of evidence for mitochondrial recombination, the standard no-recombination model was retained for the demographic analyses.

In parallel, Bayesian demographic analyses were conducted in BEAST2 (Bou et al., 2014) using the rRNA dataset together with the slowly evolving mitochondrial protein-coding genes (*cox1*, *cytb*, and *nad5*), assuming an uncorrelated lognormal relaxed molecular clock with linked clock models and a coalescent tree prior. Markov chain Monte Carlo (MCMC) analyses were run for 200 million generations, sampling every 10,000 generations, and convergence was assessed in Tracer v1.7, with all effective sample sizes (ESS) exceeding 200. Because *Pinna nobilis* is a long-lived bivalve and mitochondrial substitution rates are expected to be low in such taxa, both the substitution rate and demographic parameters were jointly estimated within the Bayesian framework. Consequently, changes in effective population size (*Ne*) are expressed as a function of the substitution rate per site per year. To estimate the timing of demographic changes, an informative prior centred on a low mitochondrial substitution rate for long-lived bivalves (μ = 1 × 10^−9^ substitutions.site^−1^.year^−1^) (Sanna et al., 2013), was applied.

#### 2.3.2 Nuclear demographic inference

To complement the mitochondrial analyses and independently assess recent demographic history, recent demographic history was inferred from nuclear genomic data using SMC++ v1.15.2. The previously filtered *P. nobilis* VCF dataset was further filtered to include only variants located on contigs longer than 1 Mb for the final analysis. The mutation rate used for time calibration was set to μ = 1 × 10^−8^ substitutions.site^−1^.generation^−1^, a value within the range of mutation rate estimates used for genomic analyses of molluscs (Li et al., 2021; Wooldridge et al., 2025). Given the absence of a directly estimated pedigree-based mutation rate for *P. nobilis*, this value was treated as an assumed calibration parameter rather than a species-specific estimates. Populations from Thau lagoon, Diane lagoon, and the open sea were analysed separately.

### 2.4 Population genetic diversity and structure analyses

Variant filtering and processing were conducted from the *P. nobilis*-only VCF dataset using VCFtools (Danecek et al., 2011) and BCFtools (Danecek et al., 2021). To minimize the influence of linkage disequilibrium among loci, biallelic SNPs were pruned using PLINK (Purcell et al., 2007) with a sliding window of 50 SNPs, a step size of 10 SNPs, and a pairwise linkage disequilibrium threshold of r^2^ = 0.2. The resulting dataset was subsequently thinned by retaining a single SNP every 500 bp using the--thin 500 option in VCFtools to reduce the density of closely spaced variants. Coding sequence coordinates were extracted and intersected with SNP positions using bedtools v2.30 (Quinlan and Hall, 2010), retaining only SNPs located outside annotated coding regions. Genome scan for selection was performed using a principal component–based genome scan implemented in the R-package pcadapt to detect loci showing strong associations with population structure (Luu et al., 2017). The purpose of this analysis was to exclude candidate loci potentially affected by selection before estimating population genetic parameters. P-values were adjusted using the Benjamini–Hochberg procedure, and loci with a false discovery rate (FDR) < 0.05 were considered candidate outlier loci and were excluded from subsequent analysis aimed at generating a putatively neutral dataset. Because only a single genome-scan method was applied, loci potentially detectable using alternative approaches may not have been identified.

Genetic diversity, including observed heterozygosity (H_O_), expected heterozygosity (H_E_), and allelic richness, as well as population genetic structure, including Wright’s inbreeding coefficient (F_IS_) and Weir and Cockerham’s F_ST_, were estimated from the putatively neutral SNP dataset after sequential filtering to remove loci with >5% missing genotypes, exclude individuals with >20% missing data, and retain only loci with a minor allele frequency (MAF) >1%. Genetic diversity indices were calculated using the R-package adegenet (Jombart and Ahmed, 2011), whereas F_IS_ and F_ST_ estimates were computed with hierfstat (Goudet, 2005). Ninety-five percent confidence intervals for F_IS_ and F_ST_ were obtained by bootstrapping across loci (1,000 replicates).

### 2.5 Genome-wide and local ancestry analyses

Model-based clustering analyses were conducted globally using ADMIXTURE software (Alexander et al., 2009), which estimates genome-wide ancestry proportions, and locally using RFMIX software (Maples et al., 2013), which infers ancestry at the chromosomal level. As these methods rely on different statistical frameworks and analytical objectives, they provide complementary insights into admixture and are not expected to produce strictly identical ancestry estimates. Analyses were performed using the filtered VCF dataset containing all samples.

#### 2.5.1 Global admixture analysis

The VCF dataset was converted to PLINK binary format (BED/BIM/FAM) using PLINK v2.0. Then, a SNP pruning was performed using a sliding-window approach with parameters adapted to increasing the detection of low-level admixture (window size = 500 SNPs, step size = 50 SNPs, r^2^ threshold = 0.2). A final LD-pruned dataset was generated by extracting the retained SNPs identified during the pruning procedure. The LD-pruned dataset was subsequently converted into additive allele format using PLINK2 (--recode A) to facilitate analyses requiring allele dosage data. For genome-wide admixture analyses, K values ranging from 2 to 5 were tested. K = 2 represented the simplest biologically interpretable model given the inclusion of two species, while higher K values were explored to assess the possibility of additional genetic structure and more complex admixture patterns. Model performance was evaluated using cross-validation error (CV), and the resulting clustering patterns were assessed in relation to the level of genetic differentiation among inferred subgroups (F_ST_).

#### 2.5.2 Local admixture analysis

Local ancestries were inferred using RFMIX software (Maples et al., 2013). Prior to local ancestry inference, the filtered VCF dataset was phased using BEAGLE (Browning et al., 2021) and subsequently thinned using VCFtools (--thin 500) to retain a maximum of one SNP every 500 bp, reducing marker redundancy while maintaining informative haplotype blocks for RFMix analyses. Individuals 1532 (GeneBank accession number GCA_054095625) and S9 were used as the reference individuals for *Pinna rudis*, while the individuals G8 (sample from Greece) and S3 (sample from Spain) were used as the reference individuals for *Pinna nobilis* species. These *P. nobilis* reference individuals were selected based on their low levels of heterozygosity, as estimated from k-mer analyses performed with Jellyfish and GenomeScope (Marcais and Kingsford, 2012; Ranallo-Benavidez et al., 2020). Because no species-specific genetic map was available, an artificial genetic map was generated directly from the VCF dataset by assigning genetic positions proportional to the physical coordinates of the variants. This artificial map was used as the recombination map required by RFMix for local ancestry inference.

#### 2.5.3 Candidate introgressed hotspots

Introgression hotspots were defined as genomic regions in which introgression was detected in more than 10% of individuals and encompassed more than 20 SNPs, in the Thau lagoon, Diane lagoon, and Open-sea populations. Introgression hotspots located on the same contig and separated by less than 10 kb were merged into a single region. Introgression hotspot statistics therefore represent recurrent introgression signals at the population level and should not be interpreted as estimates of the number or length of candidate introgressed tracts carried by individual genomes.

#### 2.5.4 DAPC analysis of admixture patterns

To summarize patterns of admixture across candidate introgressed genomic regions, a discriminant analysis of principal components (DAPC) was performed using the adegenet R package (Jombart and Ahmed, 2011). The analysis was based on admixture dosage values calculated for the candidate introgressed regions and was performed independently of sampling population. The number of genetic clusters used as groups in the DAPC was assessed using the Bayesian Information Criterion (BIC), as implemented in adegenet (Jombart and Ahmed, 2011). A coarse screen across K = 2, 3, 5, and 8 was used to evaluate increasing levels of structure and assess the consistency and interpretability of the resulting clustering patterns, rather than an exhaustive scan across all candidate K values. Among the tested values, K = 8 yielded the lowest BIC and was retained as the clustering solution for the DAPC. Because BIC did not show a clear inflection point within the tested range, K = 8 should be interpreted as the solution with the lowest BIC among those evaluated rather than as a global optimum across all possible K values. Principal component analysis was then used to reduce the dimensionality of the admixture-dosage dataset. The number of principal components retained for the DAPC was determined by cross-validation, which identified 42 principal components as the optimal number to retain. These 42 principal components were subsequently used in the discriminant analysis to characterize major patterns of variation in admixture dosage across individuals and introgressed regions.

### 2.6 Functional enrichment analysis of candidate admixed genomic regions

Genes located within candidate admixed genomic regions were identified by intersecting the coordinates of admixed intervals with annotated gene models from the reference genome. Functional annotations were obtained from InterProScan Gene Ontology (GO) assignments. Only genes with at least one GO annotation were included in the enrichment analysis, while all GO-annotated genes in the genome constituted the background set. GO enrichment analyses were performed using the Python package GOATOOLS v1.5.1 (Klopfenstein et al., 2018), employing the GO Basic ontology with propagated GO annotations and Fisher’s exact tests. Multiple testing correction was applied using the Benjamini–Hochberg false discovery rate (FDR) and GO terms with FDR-adjusted P < 0.05 were considered significantly enriched. To improve biological interpretability, root GO terms and terms outside ontology levels 1–12 were excluded from downstream analyses. Genes associated with enriched GO terms were identified and summarized by genomic contig to characterize the functional composition of candidate admixed regions.

### 2.7 Detection of genomic regions under local adaptation and recent positive selection

The genome scan was specifically designed to detect recent hard selective sweeps associated with local adaptation in the Thau lagoon population. Consequently, analyses were performed using the phased and thinned *P. nobilis*-only VCF dataset generated after quality filtering, with SNPs retained at a maximum density of one variant every 500 bp (--thin 500). The analysis focused on the joint occurrence of elevated genetic differentiation, reduced nucleotide diversity, and negative Tajima’s *D*, which together constitute the expected genomic signature of recent positive selection. Pairwise F_ST_ and nucleotide diversity (π) were estimated using VCFtools in sliding windows of 50 kb with a step size of 10 kb. Tajima’s D was calculated using the same sliding-window approach to ensure direct comparability among summary statistics. This analysis was therefore restricted to genomic patterns consistent with recent strong sweeps, and other forms of selection were beyond the scope of the present study.

Pairwise F_ST_ values were calculated between the Thau lagoon population and the two reference populations (Diane lagoon and the Open-sea), together with nucleotide diversity (π) and Tajima’s *D* for each population. For each genomic window, the minimum pairwise differentiation involving the Thau population was defined as F_ST,min_ = min (F_ST, Thau–Diane_, F_ST, Thau–Open-sea_), thereby identifying regions consistently differentiated from both reference populations.

Genome-wide outliers of F_ST,min_ were used to identify candidate regions of local adaptation. Windows with F_ST,min_ values within the upper 1% of the empirical distribution were considered candidate regions exhibiting elevated genetic differentiation between the Thau population and both reference populations. To identify high-confidence candidate regions under recent positive selection, these F_ST,min_ outlier windows were further required to exhibit reduced nucleotide diversity (i.e., π within the lower 5% of the empirical distribution) and negative Tajima’s *D* (i.e., lower 5%) in the Thau population.

Adjacent candidate windows were merged into continuous genomic regions when separated only by the sliding-window increment. To increase the robustness of candidate-region detection and reduce the influence of stochastic or localized outlier signals, only regions supported by at least three consecutive candidate windows were retained for downstream annotation and biological interpretation. Requiring a signal across consecutive overlapping windows was intended to identify spatially coherent genomic patterns rather than isolated statistical outliers.

Functional enrichment analysis was performed as described above.

### 2.8 Data accessibility

Raw sequencing reads are available from the NCBI Sequence Read Archive under BioProject accession numbers PRJNA1527659.

## 3 Results

### 3.1 DNA sequencing and variant filtering

Average number of sequencing reads was 62,072,427 (SD = 16,510,421) for the nuclear genome and 73,370 (SD = 39,736) for the mitochondrial genome, consistent with an approximate sequencing depth of 10x. Variant calling identified 104,900,089 nuclear variants across all samples, including hybrids and *P. rudis* (102,954,258 SNPs, of which 7,519,504 were multiallelic; 1,945,831 indels). Filtering retained 50,069,828 biallelic SNPs, of which 1,694,043 fell within coding sequences. Thinning (--thin 500) produced a final dataset of 1,078,378 SNPs.

### 3.2 Mitochondrial genetic diversity and structure

Across both dataset partitions (*16S rRNA* and PCG) and all sampling locations, haplotype diversity (*H*_*d*_) was uniformly high (0.932–1.000) while nucleotide diversity (Π) remained low (0.00286–0.00424). For PCG, synonymous substitutions (dS = 126–364) markedly exceeded non-synonymous substitutions (dN = 14–68) across all locations, indicating strong purifying selection (Table 2). Genetic diversity was similar across sites, with no geographic structuring (non-significant Φ_ST_). At the individual gene level (*cox1*, *cytb*, *nad5*), high *H*_*d*_ and low Π were likewise observed, with *cytb* and *nad5* showing higher polymorphism than and *cox1* (Supplementary Table S1).

**TABLE 2:** Genetic diversity and neutrality test statistics at mitochondrial markers (*16S rRNA*) and concatenated protein-coding genes (PCG) across populations (Thau lagoon, Diane lagoon and open-sea population).

​	*16S rRNA*				Concatenated PCG			
​	Thau	Diane	Open sea	All	Thau	Diane	Open sea	All
Length (nt)	1358	1358	1358	1358	4413	4413	4413	4413
N	267	37	42	345	267	31	41	345
h	118	24	31	148	209	31	35	265
Hd	0.95	0.932	0.979	0,9524	0,9973	1	0.989	0,9976
S	141	37	43	162	432	150	140	503
dS	nd	nd	nd	nd	364	134	126	424
dN	nd	nd	nd	nd	68	16	14	79
k	3.86	3.883	5.015	3.981	16.329	18.508	18.59	16.832
Π	0.00286	0.00288	0.00372	0.00295	0.00372	0.00422	0.00424	0.00383
Fu and Li’s D (overall)	−9.30482	−2.98165	−2.81147	−9.34553	−7.39614	−3.23552	−2.49547	−8.55283
P-VALUE (overall)	P < 0.02	P < 0.05	P < 0.05	P < 0.02	P < 0.02	P < 0.05	P < 0.05	P < 0.02
Tajima’s D (overall)	−2.55907	−1.9892	−1.78901	−2.56676	−2.41187	−1.94408	−1.58864	−2.42861
Tajima’s D (synonymous sites)	nd	nd	nd	nd	−2.33043	−1.84587	−1.50257	−2.35538
Tajima’s D (non-synonymous sites)	nd	nd	nd	nd	−2.33043	−2.40656	−1.99176	−2.64866
Theta	1,774	2,545	1,861	1,907	5,767	6,671	6,329	5,976
Tau	2,055	1,338	3,154	2,074	10,562	11,836	12,261	10,857
P-VALUE (overall)	P < 0.001	P < 0.05	0.05 < P < 0.1	P < 0.001	P < 0.01	P < 0.05	0.05 < P < 0.1	P < 0.01
P-VALUE (synonymous sites)	nd	nd	nd	nd	P < 0.01	P < 0.05	P > 0.1	P < 0.01
P-VALUE (non-synonymous sites)	nd	nd	nd	nd	P < 0.001	P < 0.01	P < 0.05	P < 0.001

Number of analysed sequences (N); number of haplotypes (h); haplotype diversity (Hd); number of segregating sites (S); number of synonymous and non-synonymous substitutions, (dS and dN, respectively); average number of nucleotide differences (k); nucleotide diversity (Pi); neutrality test statistics with corresponding-value (Fu and Li’s *D* and Tajima’s *D*); parameters for mutation and demographic expansion (Theta and Tau, respectively).

Haplotype networks for *16S rRNA* and *cox1* showed star-like topologies, a few high-frequency central haplotypes surrounded by many rare derived ones, and confirmed that Thau harbours diversity comparable to Diane and open-sea populations (Figure 2). Adding public NCBI haplotypes (split by pre-/post-2018 sampling) gave simpler networks, owing to shorter sequence length, but the same pattern held: Thau retains most protein-coding diversity, while several pre-2018 *16S rRNA* haplotypes are no longer detected in Thau or Diane (Supplementary Figure S2), consistent with severe demographic bottlenecks.

**FIGURE 2:**
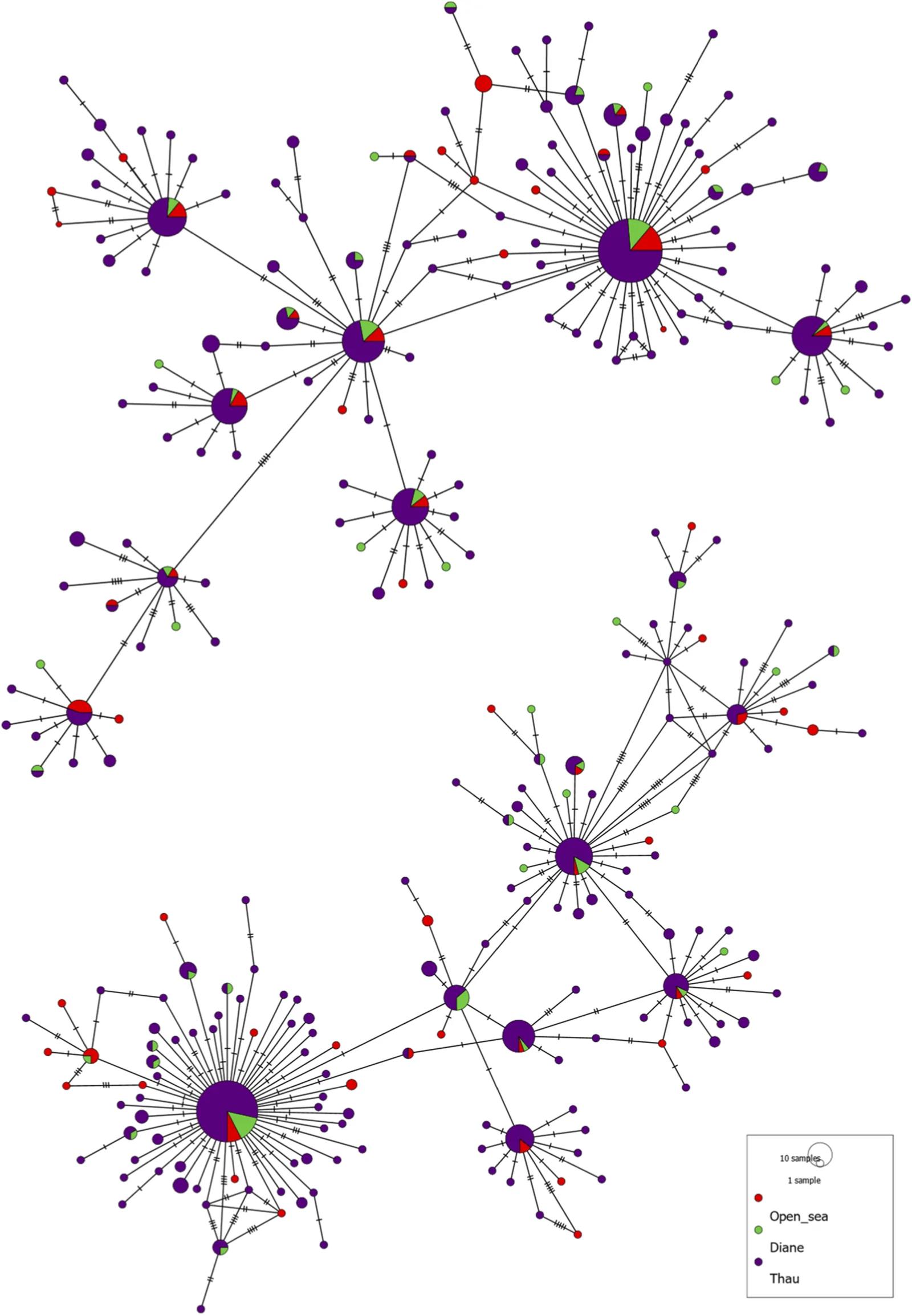
Haplotype networks of *16S rRNA* (top) and *cox1* (bottom) using full-length sequences.

All five first-generation hybrids from Spain and Diane carried *P. nobilis* mitogenomes, indicating that hybridization likely involved *P. nobilis* oocytes and *P. rudis* sperm, suggesting an asymmetric introgression. Heteroplasmy was detected in a single *P. nobilis* individual (sample 1452) among 379 individuals analysed (2.4 ^0^/_00_) and extended across the entire mitogenome. The mean read depths for *P. nobilis*- and *P. rudis*-derived sequences were 1055.07 (sd 81.29) and 85.34 (sd 18.28), respectively. The low level of nuclear admixture in this individual (0.23%) does not support a recent hybridization event. The presence of the *P. rudis*-derived mitochondrial sequence could potentially reflect historical introgression or paternal leakage, although the available data do not allow these possibilities to be distinguished. No other sample showed partial heteroplasmy indicative of recombination.

### 3.3 *Pinna nobilis* genetic diversity and structure using neutral SNPs

After data filtering, 194,964 putatively neutral SNPs were retained. Observed heterozygosity was uniformly high across groups (mean = 0.154, SD = 0.016), and inbreeding coefficients (F_IS_) were low, significant, and mostly negative, indicating a slight excess of heterozygosity. Nucleotide diversity was comparable across groups (Table 3).

**TABLE 3:** Genetic diversity statistics for each sampled populations.

​	Thau lagoon										Diane lagoon	Open sea			Overall
​	QUI	TAR	EB	BZ	SET	MRS	BAR	MEZ	MAL	BAL		Greece	Italy	Spain	
N	30	30	38	20	35	29	33	39	28	19	32	12	14	12	371
A_R_	1.358	1.357	1.357	1.352	1.353	1.353	1.351	1.352	1.354	1.354	1.352	1.332	1.295	1.348	1.348
H_o_	0.154	0.154	0.157	0.154	0.154	0.155	0.155	0.154	0.155	0.154	0.143	0.157	0.155	0.151	0.152
H_e_	0.147	0.147	0.146	0.147	0.147	0.147	0.147	0.147	0.147	0.147	0.134	0.145	0.144	0.14	0.147
F_IS_	−0.0423	−0.0331	−0.0421	−0.0364	−0.0342	−0.0366	−0.0336	−0.0358	−0.0337	−0.0334	−0.0471	−0.0101	−0.1214	−0.039	−0.0402

Sample size (N), allelic richness (A_R_), observed heterozygosity (H_o_), expected heterozygosity (H_e_), and inbreeding coefficient (F_IS_) are reported for each population.

Within Thau, pairwise F_ST_ (Table 4) among sites was extremely low and not significant (mean = 0.00061; range between 0 and 0.00219), indicating genetic homogeneity across the lagoon. Thau-vs-Open-sea and Thau-vs-Diane comparisons were significant (p-value < 0.05) but still low (F_ST_ < 0.014), consistent with limited lagoon–Open-sea connectivity; Thau was genetically closer to Spain than to Italy or Greece, a pattern also seen for Diane. Among Open-sea populations, all pair-wise F_ST_ were significant (p-value < 0.05) and reached up to 0.026, indicating shallow but clear structure.

**TABLE 4:** Pairwise genetic differentiation among *Pinna nobilis* populations based on genome-wide neutral SNPs.

​	Pairwise weighted F_ST_			
​	Mean	SD	Min	Max
Sites within Thau lagoon	0.00062	0.00053	−0.00024	0.00219
Sites within Thau lagoon vs. Greece population	**0.01388**	0.00083	**0.01328**	0.01467
Sites within Thau lagoon vs. Italy population	**0.01117**	0.00108	0.00977	0.01244
Sites within Thau lagoon vs. Spain population	0.00450	0.00108	0.00405	0.00565
Sites within Thau lagoon vs. Diane lagoon	0.00402	0.00038	0.00360	0.00456
Diane lagoon vs. Greece population	**0.01627**	​	​	​
Diane lagoon vs. Italy population	**0.01365**	​	​	​
Diane lagoon vs. Spain population	0.00773	​	​	​
Greece population vs. Italy population	**0.02671**	​	​	​
Greece population vs. Spain population	**0.01620**	​	​	​
Spain population vs. Italy population	**0.01871**	​	​	​

Pairwise weighted F_ST_, values were estimated from 194,964 neutral SNPs, among *Pinna nobilis* populations sampled from Thau lagoon, Diane lagoon, Greece, Italy, and Spain. Values for which the 95% bootstrap confidence interval excluded zero are shown in bold.

### 3.4 Demographic history

Neutrality tests were consistently negative and significant across datasets: Tajima’s *D* ranged from −1.8 to −2.6 by locus (−2.56 in the combined dataset), and Fu and Li’s *D* was similarly negative (P < 0.05–0.02) across markers. Negative values of these statistics can be consistent with recent demographic expansion but may also result from other evolutionary processes, including purifying selection or selective sweeps. The combination of high haplotype diversity and low nucleotide diversity is compatible with a recent demographic expansion, although a recent colonization or founder event cannot be excluded, particularly given the enclosed nature of the Thau lagoon (Table 2).

Mismatch distributions based on *16S rRNA* were unimodal and closely tracked the growth-decline model expectations across all populations (Supplementary Figure S3, left), which is quantitatively supported by non-significant Harpending’s raggedness (r) and sum of squared deviations (SSD) values (p-value > 0.05 for all sites). In contrast, mismatch distributions derived from the concatenated protein-coding genes were less regular. Thau lagoon showed a minor secondary shoulder (r = 0.0011; SSD = 0.01258; p-value < 0.001), Diane lagoon exhibited pronounced oscillation (r = 0.0066; SSD = 0.01083; p-value = 0.006), and Open-sea showed two subsidiary peaks flanking the main mode (r = 0.0232; SSD = 0.02345; p-value = 0.003). These significant raggedness and SSD statistics indicate that the coding-gene mismatch distributions deviate from the smooth distribution expected under a growth-decline model, suggesting a more complex or heterogeneous demographic signal than that captured by 16S rRNA alone (Supplementary Figure S3, right). Rather than contradicting the expansion signal inferred from *16S rRNA*, the greater irregularity in the protein-coding distribution is likely consistent with the multi-phase history recovered independently from genome-wide SNPs below (Figure 3).

**FIGURE 3:**
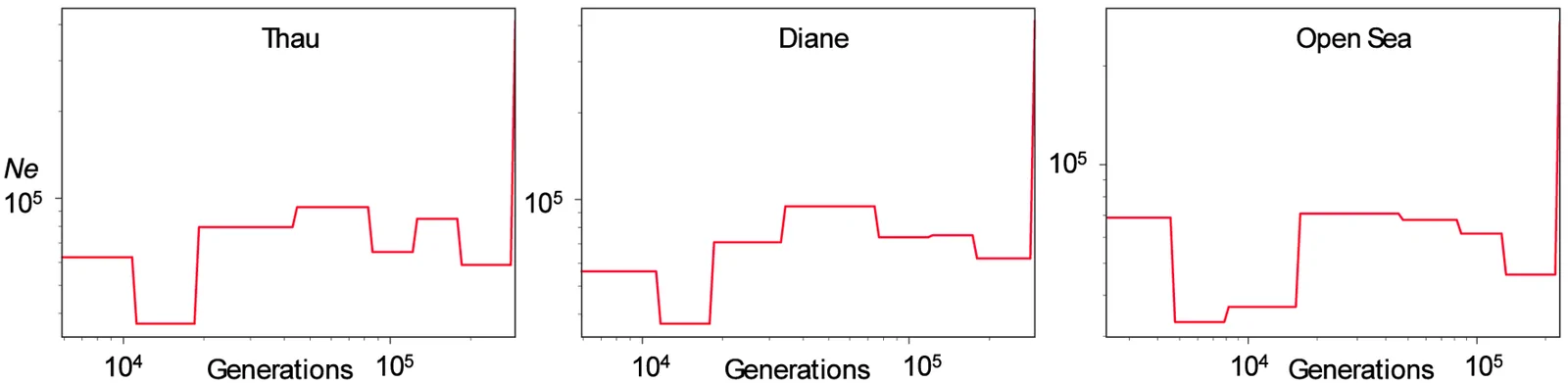
Demographic fluctuations in effective population size through time assessed with genomic SNPs. Effective population size (*Ne*) is shown as a function of generation times.

Coalescent simulations, using BEAST2 (Bou et al., 2014), dated a past expansion to approximately 800 kya in Diane and Open-sea, and 500 kya in Thau, using *16S rRNA* and assuming a mutation rate 1 × 10^−9^ site^−1^ year^−1^ (approx. 400,000–250,000 generations at a 2-year generation time). Protein-coding gene estimates (mutation rate = 3 × 10^−9^) placed the same event at roughly 250 kya (approx. 125,000 generations); together the two markers bracket a mid-to-late Pleistocene expansion (approx. 250–800 kya) (Supplementary Figure S4).

Genome-wide SNP-based simulations (mutation rate 10^−8^ site^−1^ generation^−1^), using SMC++, resolved more recent dynamics (Figure 3): *N*_*e*_ rose slightly between roughly 200,000 and 20,000 years ago, then declined sharply before a rapid post-bottleneck expansion between roughly 30,000 and 3,000 years ago. Together, mitochondrial and nuclear markers indicate a multi-phase demographic history: a mid-Pleistocene expansion, a late-Pleistocene bottleneck, and a post-glacial (late Pleistocene-Holocene) recovery.

### 3.5 Admixture analyses

Global ancestry analyses (ADMIXTURE) were performed for K = 2–5. Cross-validation error was lowest at K = 2 (0.098) wheras higher K values subdivided clusters without clear biological meaning (CV > 0.132). We therefore retain K = 2, corresponding to the two species *P. nobilis* versus *P. rudis*. At this level, ADMIXTURE identified 5 F1-hybrids, 20 individuals with 2.95%–13.43% admixture, 14 with low admixture proportions (0.04%–0.6%), and 339 individuals with no detectable admixture (Figure 4; Supplementary Table S2). Overall, considering individuals with admixture proportions above 2.9%, approximately 7% of samples were classified as admixed, with marked geographic asymmetry: 7 individuals were detected in the Thau Lagoon (Eaux-Blanches, EB; and Barrou, BAR), representing 2.3% of the local samples, 5 were identified in the Diane Lagoon (DI; 15.15%), 7 in Italy (OS; 50%), and 3 in Spain (OS; 25%, excluding F1 hybrids). Admixed Thau individuals were all located near the lagoon’s open-sea connection, indicating that the wider Thau population is predominantly non-admixed relative to Diane and Open-sea sites.

**FIGURE 4:**
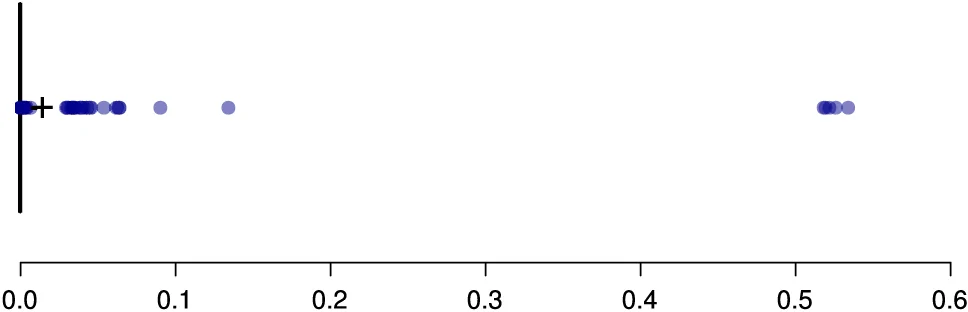
ADMIXTURE-estimated *Pinna rudis* ancestry proportion in the *Pinna nobilis* genome. Ancestry proportions were estimated for 379 individuals using 1,078,378 thinned SNPs and the ADMIXTURE software, assuming K = 2. Each point represents a single individual, and the x-axis indicates the estimated *P. rudis* ancestry proportion. Two *P. rudis* individuals with estimated ancestry proportions of 0.99999 were excluded from the figure for clarity. The cross indicates the mean estimated ancestry proportion.

Local ancestry (RFMix) told a different story as all *P. nobilis* individuals carried admixture signatures. Of 11,829 genomic windows, 4,839 (41%) showed admixture in at least one individual; individuals averaged 609 candidate admixed regions (SD 84.6), consistent with roughly 2% of the genome.

Admixture hotspots (>10% frequency in ≥1 population; n = 269) were highly shared across populations: 168 (62.5%) occurred in all three, 209 (77.7%) in at least two (Jaccard similarity 0.71–0.76), and hotspot frequencies were strongly correlated between populations (Spearman’s ρ 0.70–0.86, p < 0.001), with greater overlap than expected by chance (permutation p < 10^−4^). Over half of the hotspots occurred in more than 18% of individuals within their population (i.e. 54 individuals in Thau), indicating recurrent, non-random introgression rather than rare events (Figure 5).

**FIGURE 5:**
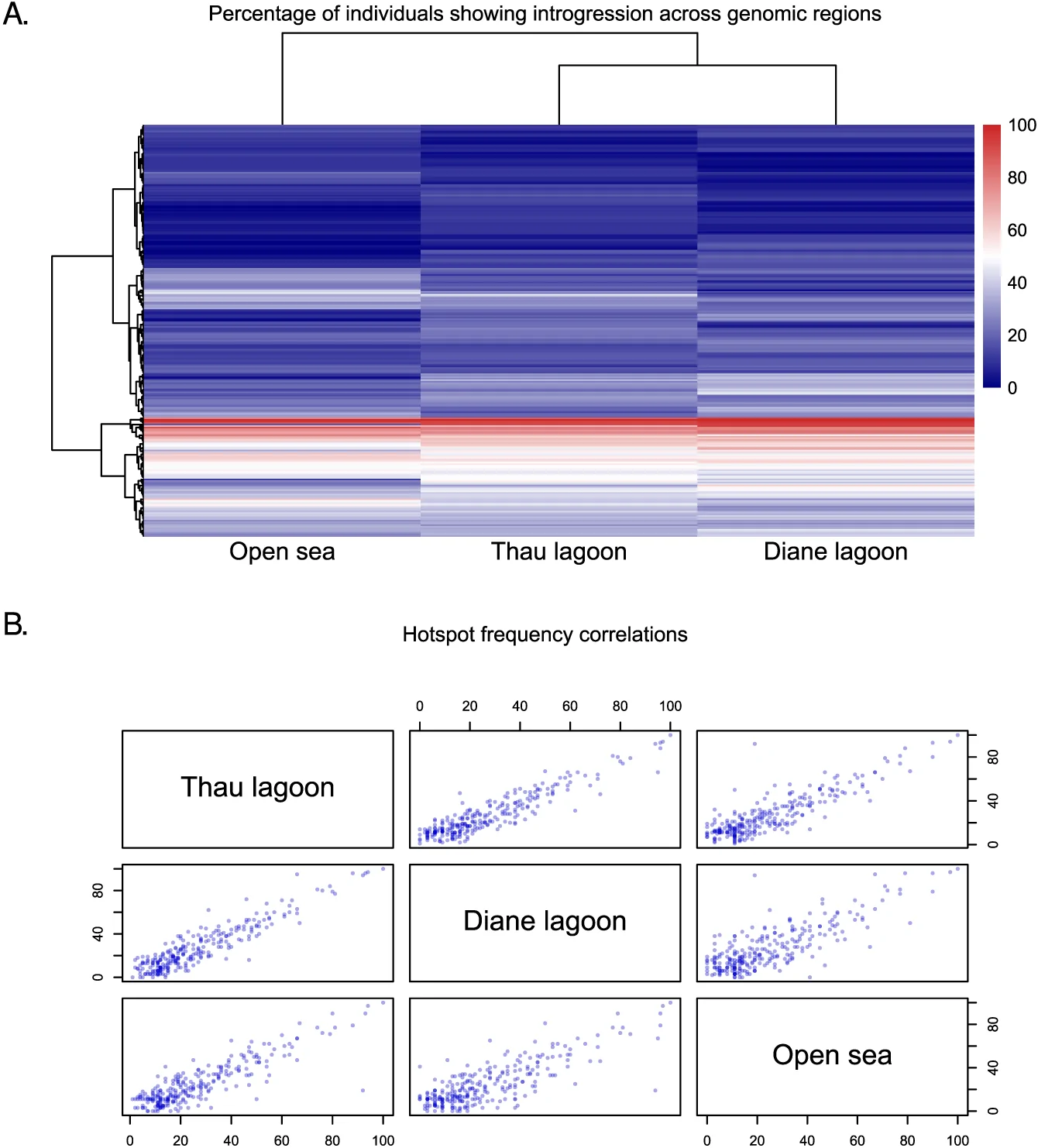
Genome-wide patterns of introgression frequency by region. **(A)** Heatmap showing the percentage of individuals showing introgression across genomic regions. Each row corresponds to a genomic region identified as an introgression hotspot, and each column represents a population. A region was defined as an introgression hotspot if introgression was detected in more than 10% of individuals in at least one population and the region contained a minimum of 20 admixed SNPs. **(B)** Pairwise correlations of introgression frequencies among the Thau lagoon, Diane lagoon, and Open-sea populations across genomic regions.

Genes within hotspots shared by >18% of individuals were enriched across all three populations for cell–cell and anchoring junctions, protein-folding chaperones, and scavenger/cargo receptor activity, with Thau additionally enriched for cell-adhesion processes and Diane for receptor-mediated functions; open-sea individuals were depleted for small-molecule metabolic genes (Table 5). Functionally, genes located within the shared candidate hotspots of introgression included multiple genes involved in innate recognition (Toll-like receptors, TRAF3-like adaptors, scavenger receptors), effector regulation (apoptosis/autophagy genes including IAPs, ULK2-like, DRAPER-like), intercellular signalling (innexins, GPCRs), epithelial adhesion/ECM (nephrin-like, cadherin-like), and stress/proteostasis (HSPs, ubiquitin ligases, PARP-family, DNA/RNA processing enzymes), forming a functionally connected set of biological pathways potentially relevant to host defence and cellular stress responses (Supplementary Table S3).

**TABLE 5:** Gene Ontology (GO) enrichment analysis of genes located within introgression hotspots shared by more than 20% of individuals in each population.

GO id	Name space	Description	Thau		Diane		OPEN_SEA	
			Fold enrichment	FDR	Fold enrichment	FDR	Fold enrichment	FDR
GO:0098609	BP	Cell-cell adhesion	3.334	1.588e-02	—	—	—	—
GO:0007155	BP	Cell adhesion	2.924	8.002e-03	—	—	—	—
GO:0044281	BP	Small molecule metabolic process	—	—	—	—	0.229	2.018e-02
GO:0005044	MF	Scavenger receptor activity	5.063	8.844e-03	7.211	5.798e-06	4.769	2.018e-02
GO:0038024	MF	Cargo receptor activity	4.778	1.190e-02	6.804	9.121e-06	4.500	2.424e-02
GO:0140662	MF	ATP-dependent protein folding chaperone	4.745	8.002e-03	4.955	4.004e-03	4.469	2.018e-02
GO:0044183	MF	Protein folding chaperone	4.459	1.190e-02	4.657	6.982e-03	4.199	2.424e-02
GO:0140657	MF	ATP-dependent activity	2.241	3.122e-02	2.260	3.898e-02	—	—
GO:0008270	MF	Zinc ion binding	1.658	2.804e-02	1.652	4.606e-02	—	—
GO:0003690	MF	Double-stranded DNA binding	0.184	4.010e-02	—	—	0.174	3.710e-02
GO:0003700	MF	DNA-binding transcription factor activity	0.179	3.122e-02	—	—	—	—
GO:0005911	CC	Cell-cell junction	6.364	2.269e-04	6.135	8.445e-04	5.532	5.027e-03
GO:0070161	CC	Anchoring junction	5.346	1.154e-03	5.153	3.369e-03	4.647	1.690e-02
GO:0030054	CC	Cell junction	3.821	1.190e-02	—	—	—	—
GO:0110165	CC	Cellular anatomical structure	0.782	3.752e-04	0.801	3.583e-03	—	—
GO:0005575	CC	cellular_component	0.746	4.135e-08	0.748	1.509e-07	0.817	2.275e-05
GO:0032991	CC	Protein-containing complex	0.571	2.804e-02	0.505	3.369e-03	—	—

GO, enrichment was performed using GOATOOLS, with all GO-annotated genes as the background. Fold enrichment values greater than 1 indicate GO, categories that are overrepresented among introgressed genes relative to the genome-wide background, whereas values below 1 indicate underrepresented categories. Only GO, terms with a false discovery rate (FDR) < 0.05 are shown.

DAPC based on genome-wide local ancestry dosage was used to characterize clustering according to introgression profiles. At K = 2, two well-separated groups were identified along LD1, while increasing K progressively resolved additional components without disrupting the major grouping. The K = 3–8 solutions revealed three consistently recovered clusters together with several smaller, partially overlapping components (Figure 6). We retained the K = 8 solution as the highest resolution shown because further increases in K produced only minor subdivisions and did not substantially alter the underlying DAPC structure (data not shown). The persistence of the major clusters across K values indicates that the observed structure reflects shared patterns of local ancestry among individuals rather than an artefact of a particular clustering resolution.

**FIGURE 6:**
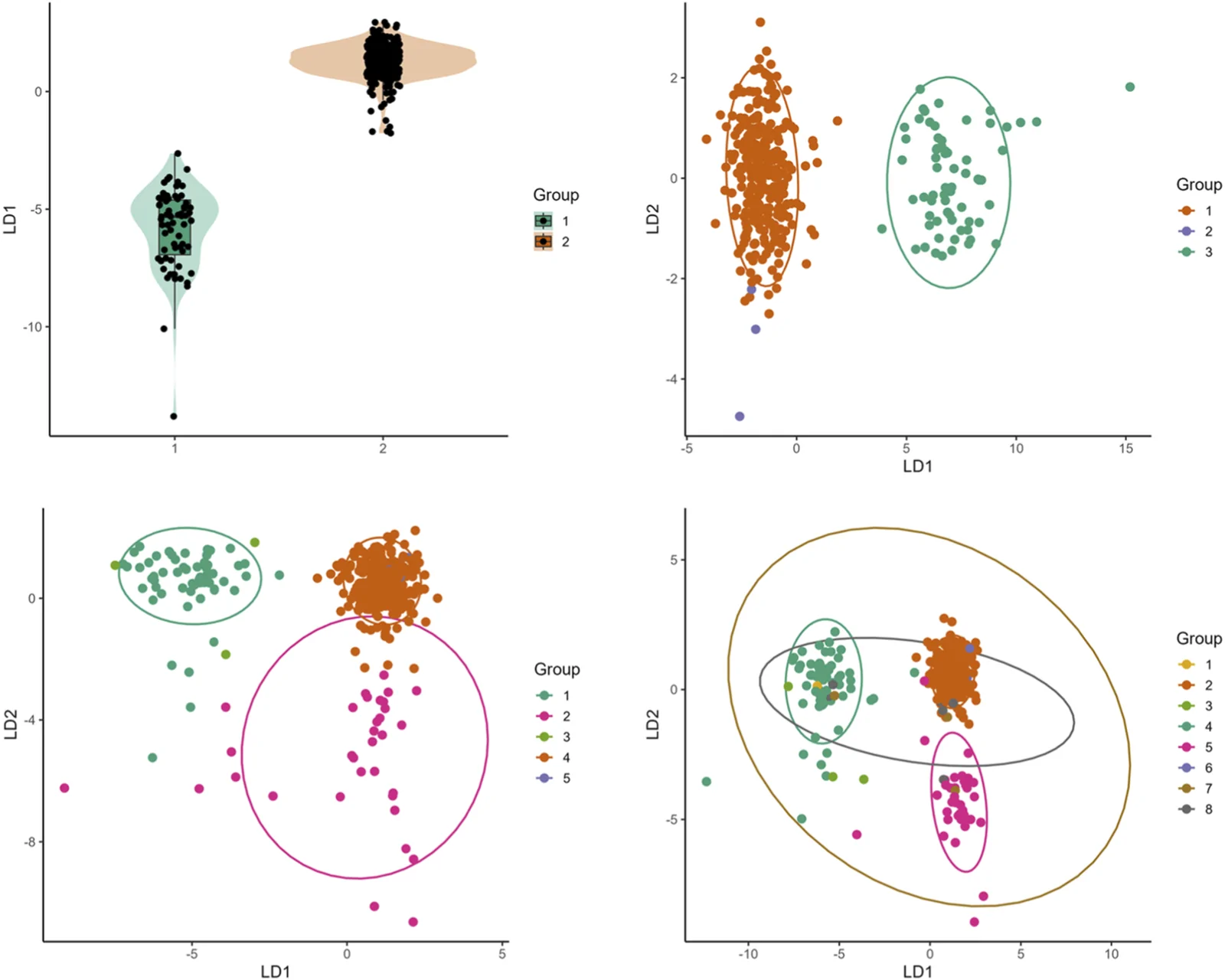
Hierarchical genetic structure inferred from DAPC based on local ancestry dosage data. Discriminant Analysis of Principal Components (DAPC) was applied to genome-wide local ancestry dosage data inferred using RFMix (0, 1, or 2 ancestry copies per locus) to assess patterns of genetic clustering. Panels show increasing cluster resolution from K = 2, 3, 5 and 8. Individuals are assigned to clusters based on their local ancestry profiles, with cluster assignments in the K = 2 and K = 3 solutions represented using consistent colour coding to facilitate comparison across hierarchical levels.

At K = 8, a dominant shared component (cluster 2) accounted for 67%–69% of ancestry across all populations; cluster 4 was more common in Open-sea individuals (26% vs. 15%–19% in lagoons) while cluster 5 showed the reverse (11%–13% in lagoons vs. 5% open-sea) (Figure 7), Open-sea individuals separated along LD1, lagoon individuals along LD2.

**FIGURE 7:**
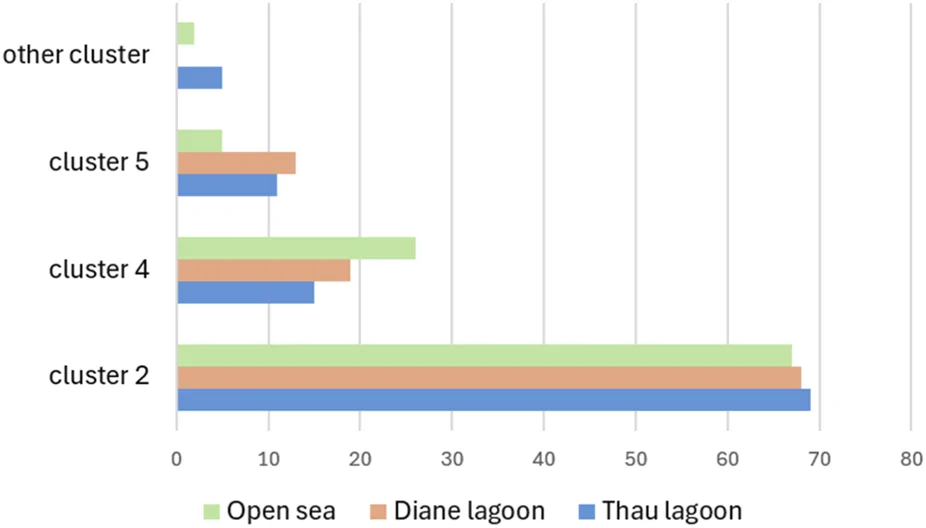
Cluster composition at k = 8 based on local ancestry dosage inferred from DAPC clustering. Proportional assignment of individuals (Thau and Diane lagoons, and Open-sea) to ancestry clusters inferred at K = 8 from Discriminant Analysis of Principal Components (DAPC) applied to genome-wide local ancestry dosage data (0, 1, or 2 ancestry copies per locus). Values represent percentage membership of each individual in each cluster.

LD1 was driven by 28 genes almost exclusively admixed in Cluster 4, spanning transcriptional regulation, RNA processing, trafficking, and protein modification (zinc-finger proteins, DMRT1, MAX, hnRNP U, SRSF6, MON1-CCZ1 components, FBXO6, farnesyltransferase β-subunit), plus mitochondrial, cytoskeletal, and transport genes (Supplementary Table S4). Cluster 5 (mainly Thau/Diane individuals) was enriched for genes involved in membrane organization, immune recognition, trafficking, and signalling including CD53-like, tetraspanin-9, LRP1, glycine transporter, fucolectins, scavenger receptor class F, SRP54, TDRD9, BCS1, YIPF4, and several kinases/phosphatases (Table 6; Supplementary Table S4). These functional differences may reflect environmental-specific variations in the retention or distribution of candidate introgressed regions, although their biological significance remains to be established.

**TABLE 6:** Annotated genes located within genomic regions showing the highest admixture frequencies in ancestry cluster 5. Annotated genes were identified within genomic regions exhibiting the highest admixture frequencies in ancestry cluster 5, which is composed predominantly of individuals from the Thau and Diane lagoons.

GeneID	Description	Coordinates
g9623	ATP-dependent RNA helicase TDRD9-like isoform X1 [*Ostrea edulis*]	Pnobilis_contig218:432,203–464165
g9608	BTB/POZ domain-containing protein KCTD3-like [Ylistrum balloti]	Pnobilis_contig218:4465–32926
g9626	Cap-specific mRNA (nucleoside-2′-O-)-methyltransferase 1-like isoform X1 [*Ostrea edulis*]	Pnobilis_contig218:727,154–753460
g9615	Dipeptidyl peptidase 3-like isoform X2 [*Mytilus galloprovincialis*]	Pnobilis_contig218:152,563–179785
g9639	Epididymal secretory protein E1-like [*Crassostrea virginica*]	Pnobilis_contig218:1,278,432–1286945
g9611	Exonuclease 1-like [Saccostrea echinata]	Pnobilis_contig218:82,775–107132
g4666	Fucolectin-1-like, partial [Ylistrum balloti]	Pnobilis_contig162:473,044–485966
g4657	Fucolectin-5-like [*Argopecten irradians*]	Pnobilis_contig162:22,233–57235
g9634	Galactose mutarotase isoform X1 [Magallana gigas]	Pnobilis_contig218:1,074,629–1087863
g9630	Galactose mutarotase-like [Magallana angulata]	Pnobilis_contig218:912,990–919333
g9631	Galactose mutarotase-like [Saccostrea echinata]	Pnobilis_contig218:921,427–930685
g9632	Galactose mutarotase-like [Saccostrea echinata]	Pnobilis_contig218:951,669–957904
g9629	H2.0-like homeobox protein [*Pecten maximus*]	Pnobilis_contig218:866,682–875308
g9614	Kelch-like protein 12 [Saccostrea echinata]	Pnobilis_contig218:139,922–141878
g1197	Leukocyte surface antigen CD53-like isoform X1 [*Mytilus edulis*]	Pnobilis_contig115:1091934–1100358
g14828	Low-density lipoprotein receptor-related protein 1-like [Magallana angulata]	Pnobilis_contig284:357,677–402478
g9618	Mitochondrial chaperone BCS1-like [Saccostrea echinata]	Pnobilis_contig218:335,788–339664
g9517	Mushroom body large-type Kenyon cell-specific protein 1-like [*Ostrea edulis*]	Pnobilis_contig216:607,251–621898
g9640	NPC intracellular cholesterol transporter 2-like [Saccostrea cuccullata]	Pnobilis_contig218:1300761–1308691
g9624	Optic atrophy 3 protein homolog [Magallana gigas]	Pnobilis_contig218:468,108–469377
g9620	Protein phosphatase 1 regulatory subunit 37 [Magallana gigas]	Pnobilis_contig218:370,527–396999
g9613	Protein YIPF6-like [*Ostrea edulis*]	Pnobilis_contig218:120,034–128674
g4661	Rieske domain-containing protein-like [Saccostrea echinata]	Pnobilis_contig162:228,978–236576
g4658	Scavenger receptor class F member 2-like isoform X3 [Mizuhopecten yessoensis]	Pnobilis_contig162:74,622–104818
g9635	Serine/threonine-protein kinase ULK4-like isoform X3 [*Ostrea edulis*]	Pnobilis_contig218:1092902–1126672
g9619	Serine/threonine-protein kinase VRK1-like [Saccostrea echinata]	Pnobilis_contig218:342,740–367524
g9609	Signal recognition particle subunit SRP54 [Saccostrea echinata]	Pnobilis_contig218:42,559–60269
g12637	Sodium- and chloride-dependent glycine transporter 1-like [Mizuhopecten yessoensis]	Pnobilis_contig263:861,686–878778
g4660	Spermine synthase-like [Saccostrea cucullata]	Pnobilis_contig162:170,282–188894
g9625	Synaptotagmin-14-like isoform X8 [*Ostrea edulis*]	Pnobilis_contig218:482,547–540750
g1198	Tetraspanin-9-like isoform X2 [*Ostrea edulis*]	Pnobilis_contig115:1109153–1119935
g9638	Triokinase/FMN cyclase-like [*Ostrea edulis*]	Pnobilis_contig218:1243123–1267665
g4665	Uncharacterized protein K02A2.6-like [Magallana angulata]	Pnobilis_contig162:413,641–417808
g9612	Uncharacterized protein LOC117343268 [*Pecten maximus*]	Pnobilis_contig218:113,099–116750
g9617	Uncharacterized protein LOC130054725 [*Ostrea edulis*]	Pnobilis_contig218:289,324–307965
g9641	Uncharacterized protein LOC130054827 [*Ostrea edulis*]	Pnobilis_contig218:1327115–1350776
g4662	Uncharacterized protein LOC134274555 [Saccostrea cuccullata]	Pnobilis_contig162:286,233–287967
g9637	Visual system homeobox 2-like [*Ostrea edulis*]	Pnobilis_contig218:1168856–1189458
g9628	YLP motif-containing protein 1 isoform X6 [Magallana gigas]	Pnobilis_contig218:787,480–835249

### 3.6 Selection scans

A total of 732 genomic windows showed local-adaptation signals in Thau; 111 regions (≥3 consecutive outlier windows), containing 255 coding sequences, were retained (Supplementary Tables S5–S7). No GO category survived multiple-testing correction, but candidate genes recurred in four categories: cellular stress/DNA repair (HSP12A/B), immune recognition (TLR4, DDX58-like, IRF1-like, NACHT-domain, apoptosis genes), solute transport/metabolism (monocarboxylate transporters, SLC family), and lipid metabolism/membrane/ECM/signal transduction.

Positive selection scans identified two genes: a procollagen lysine hydroxylase/glycosyltransferase LH3-like gene (extracellular matrix maturation) and a prostaglandin E2 receptor EP4-like gene (lipid-mediated signalling) (Supplementary Tables S5, S6, S8). Only a small number of locally adaptive regions overlapped introgression hotspots. These contained genes for membrane trafficking (PI3P 5-kinase-like), protein turnover (FBXO39 homologs), and chromosome organization (SKA2-like, TERB1-like), which are not classic stress-response genes, but suggestive that candidate introgressed haplotypes may act through regulatory rather than direct stress pathways.

## 4 Discussion

*Pinna nobilis* conservation is an active emergency. Mass mortality events (MMEs) continue to threaten the species: the parasite *Haplosporidium pinnae* remains detectable, implying either long-term persistence in the open sea or additional reservoir hosts, and other pathogens, including picornaviruses, appear to also contribute to declines (Carella et al., 2023b; Carella et al., 2026).

Remaining populations are now largely confined to paralic environments, making these populations particularly relevant for understanding the genetic diversity and persistence of the species and for evaluating their potential as sources for conservation and restoration. However, their confinement to such environments may limit connectivity with open-sea populations and constrain natural recovery. Conservation therefore increasingly relies on active interventions, such as translocation from refugial populations like Thau lagoon to open-sea habitats.

This raises a central question: do refugial populations such as Thau retain the genetic and adaptive characteristics of historical open-sea populations, making them viable sources for species recovery? Azzena et al. (2025) previously reported reduced *cox1* haplotype diversity, including loss of haplotypes from both clade A and B; our results support this pattern of erosion, though Thau appears to have largely escaped recent MMEs (Foulquié et al., 2026), raising the possibility that ancestral variants persist there. The hypothesis of adaptive introgression also merits investigation (Coupé et al., 2023; Lattos et al., 2025). Although experimental approaches such as genome editing, molecular manipulation, or controlled infection assays are currently not feasible in *P. nobilis*, our analyses identify candidate introgressed regions containing genes involved in immune processes and cellular regulation that may be relevant to variation in the MME response. These regions therefore provide promising candidates for future studies integrating genomic and phenotypic data.

### 4.1 Genetic diversity and population structure

#### 4.1.1 Mitochondrial markers

The comparison of haplotype diversity (based on NCBI makers) between historical samples collected before the MMEs and contemporary samples collected after MMEs (Supplementary Figure S2) revealed a greater reduction in the number of *16S rRNA* haplotypes (54%) than *cox1* haplotypes (33%), consistent with prior work (Azzena et al., 2025). Although the two markers showed similar numbers of haplotypes in the historical samples, differences in sampling periods and spatial coverage mean that these percentages cannot be interpreted solely as resulting from the recent MMEs. The stronger reduction observed for *16S rRNA* may instead reflect marker-specific characteristics, such as differences in mutation rates or the distribution of informative sites between the two regions. Full-length mitogenomes showed high, broadly shared diversity between Thau and Open-sea populations despite the demographic collapse, most plausibly because Thau’s exceptionally high population density buffered genetic drift during the bottleneck (Foulquié et al., 2026).

#### 4.1.2 Nuclear markers

The Thau lagoon population exhibited high levels of genetic diversity (Table 3), comparable to those reported for open-sea populations. Despite the lagoon’s semi-enclosed nature, no evidence of within-population genetic structure was detected, suggesting that the population is effectively panmictic (Table 4). The absence of detectable genetic structure within the lagoon may be explained by its hydrodynamic regime, which promotes water circulation and larval dispersal throughout the lagoon despite its limited exchange with the open sea. These findings are in agreement with previous microsatellite-based studies, which similarly reported high genetic diversity and little evidence of within-lagoon genetic structure in *P. nobilis* populations (Nebot-Colomer et al.). In contrast, significant genetic differentiation was detected between the Thau lagoon and several Open-sea populations (Table 4). The strongest differentiation was observed with northern Sardinia (Italy) and Greece (F_ST_ = 0.014 and 0.011, respectively), whereas differentiation with Spain was comparatively weaker (F_ST_ = 0.004). The pronounced differentiation between the Thau Lagoon and the Greek population is consistent with the west–east genetic structure previously reported in *P. nobilis*, with the genetic boundary between western and eastern Mediterranean populations inferred to lie east of the Sicilian Strait (Sanna et al., 2023; Sanna et al., 2013; Sarafidou et al., 2023). More unexpectedly, within the western Mediterranean, the Thau lagoon population was more differentiated from northern Sardinia than from Spain, indicating a closer genetic relatedness with Iberian populations. These findings complement previous microsatellite studies, which reported little or no genetic differentiation among populations within the western Mediterranean basin (Wesselmann et al., 2018; Peyran et al., 2021). The higher resolution afforded by genome-wide SNPs likely enabled the detection of this fine-scale population structure, revealing subtle patterns of restricted connectivity that remained undetected with lower-density genetic markers (Vendrami et al., 2017; Zimmerman et al., 2020). Although the magnitude of genetic differentiation remained low and falls within the range previously reported for nuclear markers (pairwise F_ST_ generally < 0.02) across western Mediterranean populations (Wesselmann et al., 2018; Peyran et al., 2021; Peyran et al., 2022), the significant differentiation involving the Thau lagoon indicates that this population is not fully connected to surrounding open-sea populations and likely experiences restricted gene flow. This finding is particularly noteworthy given the generally weak population structure previously described for the species using both microsatellite and genome-wide markers (Wesselmann et al., 2018; Peyran et al., 2021; Azzena et al., 2025). Collectively, these results suggest that the Thau lagoon constitutes a partially isolated genetic unit whose evolutionary and conservation significance warrants further investigation. Based on these results and taking onto account that the Thau population is still healthy (Foulquié et al., 2024; Foulquié et al., 2026), management strategies should aim to protect its genetic diversity while maintaining opportunities for genetic exchange with depleted open-sea populations. This could include monitoring and, where appropriate, managing introgression from heavily impacted or genetically distinct populations, while also considering the potential for natural or assisted gene flow to contribute to the genetic rescue of depleted populations (Whiteley et al., 2015; Fitzpatrick et al., 2020).

### 4.2 Demographic history

The inferred demographic history (Figure 3; Supplementary Figure S4), characterized by successive population contraction and expansion events, match typical Pleistocene contraction-expansion signatures in marine sessile taxa and are broadly congruent with Sanna et al.'s earlier mitochondrial-based findings (Sanna et al., 2023), now refined with genome-wide data revealing more recent dynamics. Absolute dating depends on mutation rate estimates currently unavailable for *P*. *nobilis*. Given the species’ long lifespan, the true rate is likely lower than assumed, so these results should primarily be considered as a relative framework describing the sequence and magnitude of demographic changes, while the absolute timing of events may be adjusted as more accurate mutation rate estimates become available. Moreover, the temporal limits of the genetic signal and uncertainties in molecular-rate calibration reduce the resolution of demographic fluctuations at the edges of the inferred time frame. Consequently, population size changes near these temporal boundaries reflect broad demographic trends rather than precisely dated events and should not be used to interpret recent population dynamics.

The ability of *P. nobilis* to recover from severe historical bottlenecks and regenerate genetic diversity is encouraging. However, the demographic impact appears substantial and whether the species can undergo natural demographic and genetic recovery following the recent collapse remains uncertain. If possible, future sampling of temporal cohorts from open-sea populations could directly quantify recent genetic erosion and demographic decline, allowing estimation of the ongoing reduction in effective population size. Such data would provide a valuable framework for assessing the species’ evolutionary resilience and informing conservation strategies. From a conservation perspective, these findings also highlight the potential value of preserving small remnant populations in other affected areas. Even populations with low abundance may retain relevant genetic diversity and contribute to the long-term recovery of the species, provided that they are not completely isolated and that sufficient connectivity remains to allow gene flow. Consequently, conservation efforts should not focus exclusively on large or apparently healthy populations, but should also consider the preservation of small, spatially coherent populations that may serve as reservoirs of genetic diversity and contribute to the species’ future recovery.

### 4.3 Genomic signatures of local adaptation in the Thau lagoon environment

Localised departures from neutrality (i.e., reduced diversity, elevated differentiation, strongly negative Tajima’s *D*) identify genomic regions that may have been affected by selection (Table 2). Candidate genes (Supplementary Table S7) span stress response, immune regulation, metabolism, transport, and cellular homeostasis rather than clustering in a few pathways, consistent with the polygenic local adaptation typical of marine bivalves in physiologically demanding lagoon environments (Pérez-Ruzafa et al., 2011; Derolez et al., 2020; Quero et al., 2017; Trombetta et al., 2022). The absence of GO enrichment likely reflects this dispersed architecture together with limited functional annotation for non-model species. Heat-shock, DNA-repair, and proteostasis genes point to maintenance of cellular integrity under recurrent stress, transporter genes to metabolic and ionic regulation, and lipid-metabolism and membrane genes to preserving function under thermal and osmotic flux.

Lagoon microbial exposure likely also drives selection on immune genes (TLR, DDX58-like, IRF1-like, NACHT-domain, apoptosis genes), supporting host–microbe interaction as a key selective pressure, though the specific pathogens involved cannot be identified from this data (Guo and Ford, 2016; Green and Speck, 2018; Takeuchi et al., 2022; Danovaro and Pusceddu, 2007; Gignoux-Wolfsohn et al., 2024). Two genes under positive selection, the LH3-like collagen-modification gene and the EP4-like prostaglandin receptor, point respectively to structural and tissue-repair adaptation, relevant for a sessile organism, and to lipid-signalling-mediated stress and immune regulation, complementing the broader local-adaptation signal (Supplementary Table S8). Similar patterns have been reported in other marine invertebrates, where immune- and stress-related genes are recurrently identified as targets of positive selection. For example, adaptive evolution in *Crassostrea gigas* has been associated with immune- and stress-related genes (Wendling and Wegner, 2015), while comparative genomic studies in *Mytilus* spp. have identified positive selection in genes involved in immunity, stress response, and shell formation (Figueras et al., 2019; Saco et al., 2023). These parallels suggest that immune regulation, stress response, and maintenance of structural integrity may represent recurrent targets of local adaptation in sessile marine invertebrates exposed to heterogeneous environmental and microbial conditions, although the specific genes and selective pressures may differ among species (Song et al., 2019; Zhang et al., 2012).

Although consistent with selection pressures imposed by pathogens and other environmental stressors, our results may also partly reflect demographic history, which can mimic signatures of selection through changes in genetic diversity, differentiation, and neutrality statistics (Akey et al., 2004; Nielsen, 2005). In particular, demographic expansion can generate departures from neutrality and patterns of heterogeneous diversity that overlap with those expected under selection (Excoffier and Ray, 2008). In the Thau population, recent demographic expansion is supported by our analyses, whereas the occurrence and extent of past bottlenecks cannot be established from the present data. Demographic history should therefore be considered when interpreting the candidate regions of local adaptation.

Because the Thau population has been relatively spared from MMEs, the detected signatures cannot be directly attributed to recent MME-associated disease pressure, such as that caused by *H. pinnae*. Instead, they may reflect pre-existing adaptive variation and historical or recurrent selection associated with environmental and pathogen-related stress, potentially contributing to the population’s resilience. A recent genome-wide selective sweep specifically driven by the MME outbreak is also unlikely given the limited exposure of this population to MMEs, although selection on genomic regions not captured by our scans cannot be excluded. Only a few locally adaptive regions overlapped introgression hotspots, suggesting that introgression may contribute to adaptation at a limited number of loci rather than genome wide. The overlapping genes, involved in membrane trafficking, ubiquitin-mediated turnover, and chromosome organization, suggest regulatory rather than direct stress-response roles, consistent with, but not demonstrating, adaptive introgression (Lin et al., 2026; Hage and Rajsbaum, 2019; Mukherjee and Dikic, 2022). Functional validation will be needed to assess the contribution of these candidate regions to adaptation.

### 4.4 Heteroplasmy and admixture

Although mitochondrial heteroplasmy is common in bivalves, notably those exhibiting doubly uniparental inheritance (DUI) such as *Mytilus* mussels, DUI has not been reported in *P. nobilis*, and previous mitochondrial studies have not documented clear cases of intraspecific heteroplasmy in this species (Sanna et al., 2023; Catanese et al., 2022). In DUI species, two sex-associated mitochondrial lineages are transmitted: an F-type through eggs and an M-type through sperm, producing characteristic sex-specific heteroplasmy (Breton et al., 2006; Capt et al., 2020). In our dataset, mitochondrial variants representing less than 10% of the reads were filtered out, and the remaining variants were assessed to determine whether they belonged to *P. nobilis* or *P. rudis*. No clear cases of intraspecific heteroplasmy were detected above this threshold. The heteroplasmy observed here is therefore unlikely to reflect the typical DUI system and may instead be associated with interspecific admixture.

In our samples, one Spanish *P. nobilis* individual carried mitochondrial haplotypes assigned to both *P. nobilis* and P. rudis. Read-level inspection using IGV (Thorvaldsdóttir et al., 2013) further confirmed the presence of both haplotypes across the full-length mitochondrial genome, supporting the occurrence of heteroplasmy. This observation is thus consistent with interspecific mitochondrial heteroplasmy and indicates that admixture between these species can extend to the mitochondrial genome. Given that this observation has been made only once in our sample set, further sampling will be required to precise its frequency. All five F1 hybrids carried only *P. nobilis* mitogenomes, suggesting either directional hybridization (*P. nobilis* maternal, *P. rudis* paternal) or selective loss of *P. rudis* mitochondria, a pattern future hybrid studies should track, given its potential fitness implications under pathogen and environmental stress (Hill, 2015; Burton et al., 2013), and its relevance as *P. rudis* research expands (Catanese et al., 2022; Azzena et al., 2026; Lopes et al., 2025; Fassio et al., 2025). This pattern may represent a resilience advantage, supporting the scenario of the shared historical introgression event, when ongoing gene flow among populations is present, as is the case in the Spanish population.

One important question raised by the widespread local ancestry signal detected by RFMix is whether it reflects genuine historical introgression or incomplete lineage sorting (ILS). Although both processes can generate shared genetic variation between closely related species, several observations argue against ILS as the primary explanation. Based on current estimates, *P. nobilis* and *P. rudis* diverged approximately 2 million years ago (Sanna et al., 2023). Given the effective population sizes inferred here (approximately 3 × 10^4^ to 10^5^ individuals), this divergence time is expected to be large relative to those effective population sizes when expressed in coalescent units (i.e., *T* >> 2*N*_*e*_), making the persistence of ancestral polymorphism over the inferred divergence period less likely under a neutral ILS scenario (Wakeley, 2009). Furthermore, the inferred *P. rudis* ancestry was concentrated in a limited number of genomic regions that were remarkably consistent across geographically separated populations. More than 50% of introgression hotspots were shared among all populations, and hotspot frequencies were highly correlated (Figure 5). From a biological perspective, this pattern suggests that the functional composition of candidate introgressed regions is largely shared across populations rather than being driven by population-specific processes. Such a pattern is consistent with common evolutionary dynamics acting across the species’ range, including a shared historical introgression event, ongoing gene flow among populations, or heterogeneous genomic permeability to introgression. Together, these dynamics are difficult to reconcile with stochastic lineage sorting but are readily explained by historical introgression followed by the long-term persistence of candidate introgressed haplotypes. The contrasting patterns obtained with ADMIXTURE and RFMix further support this interpretation. Whereas ADMIXTURE identified admixture only in recent hybrids and individuals carrying relatively high proportions of *P. rudis* ancestry, RFMix detected short candidate introgressed tracts in all *P. nobilis* individuals. This discrepancy is expected under ancient introgression, where recombination progressively fragments introgressed haplotypes into short genomic segments that contribute little to genome-wide ancestry proportions while remaining detectable by local ancestry inference (Liang and Nielsen, 2014; Gravel, 2012; Racimo et al., 2015). In addition, ADMIXTURE infers ancestry relative to the sampled populations and assumes that they represent the ancestral gene pools. If all extant *P. nobilis* individuals already share a similar background of ancient introgression, no truly non-admixed reference population remains, and the shared introgressed component is effectively incorporated into the baseline genetic background rather than identified as admixture (Alexander et al., 2009; Lawson et al., 2018). By contrast, RFMix exploits haplotype structure and linkage information to identify localized ancestry tracts, making it more sensitive to low levels of ancient introgression (Maples et al., 2013). Finally, the enrichment of recurrent introgression hotspots for genes involved in innate immunity, cell adhesion, stress responses and protein homeostasis (Tables 5,6; Supplementary Tables S3, S4) suggests that at least part of this shared ancestry may have been preferentially retained by natural selection (Martin and Jiggins, 2017; Durand et al., 2011).

Global admixture proportions inferred with ADMIXTURE indicated low levels of *P. rudis* ancestry in a subset of the analysed *P. nobilis* individuals, whereas other individuals showed no detectable *P. rudis* ancestry with this approach (Figure 4; Supplementary Table S2). Importantly, the low-level ancestry signals observed in *P. nobilis* are unlikely to be explained by artefacts related to missing data or rare variant effects, given the stringent data filtering applied, the low overall missingness, and the exclusion of rare alleles. In addition, the strong genetic differentiation between parental species (F_ST_ = 0.27) supports the reliability of ancestry inference. Under these conditions, the observed background admixture is more plausibly interpreted as a weak but genuine signal of historical or ongoing gene flow rather than a methodological artefact or stochastic noise. This interpretation is complementary to the RFMix results. Whereas ADMIXTURE detected genome-wide ancestry proportions only in a subset of individuals, RFMix identified short candidate *P. rudis*-like ancestry tracts across all *P. nobilis* individuals, consistent with the possibility that ancient introgressed segments are widely shared but contribute too little to genome-wide ancestry proportions to be consistently detected by ADMIXTURE.

Beyond supporting the occurrence of historical introgression, the functional composition of shared candidate introgressed regions provides clues about the potential biological relevance of these regions. A substantial fraction of the candidate *P. rudis*-like regions was shared by approximately 20% of individuals across populations and included genes involved in pathogen recognition, immune signalling, apoptosis, adhesion, and proteostasis. This functional enrichment suggests that candidate introgressed regions may contribute to biological processes involved in host defence and cellular stress responses. However, because our study does not directly compare resistant and susceptible individuals and does not test the phenotypic effects of individual introgressedF alleles, these results should be considered as identifying candidate regions potentially associated with resilience rather than evidence that introgression confers disease resistance or tolerance. The apparent coherence of these functional categories, including innate recognition (TLRs, scavenger receptors, TRAF3-like), downstream apoptosis and autophagy, tissue and adhesion genes (cadherins, nephrins, innexins, GPCRs), and proteostasis-related genes (HSPs, ubiquitin pathway, DNA/RNA repair), provides a basis for future functional investigation (Figure 8). Population-specific differences, such as adhesion enrichment in Thau and receptor-mediated enrichment in Diane, may reflect differences in the distribution of candidate introgressed regions among populations, although their functional significance remains to be determined.

**FIGURE 8:**
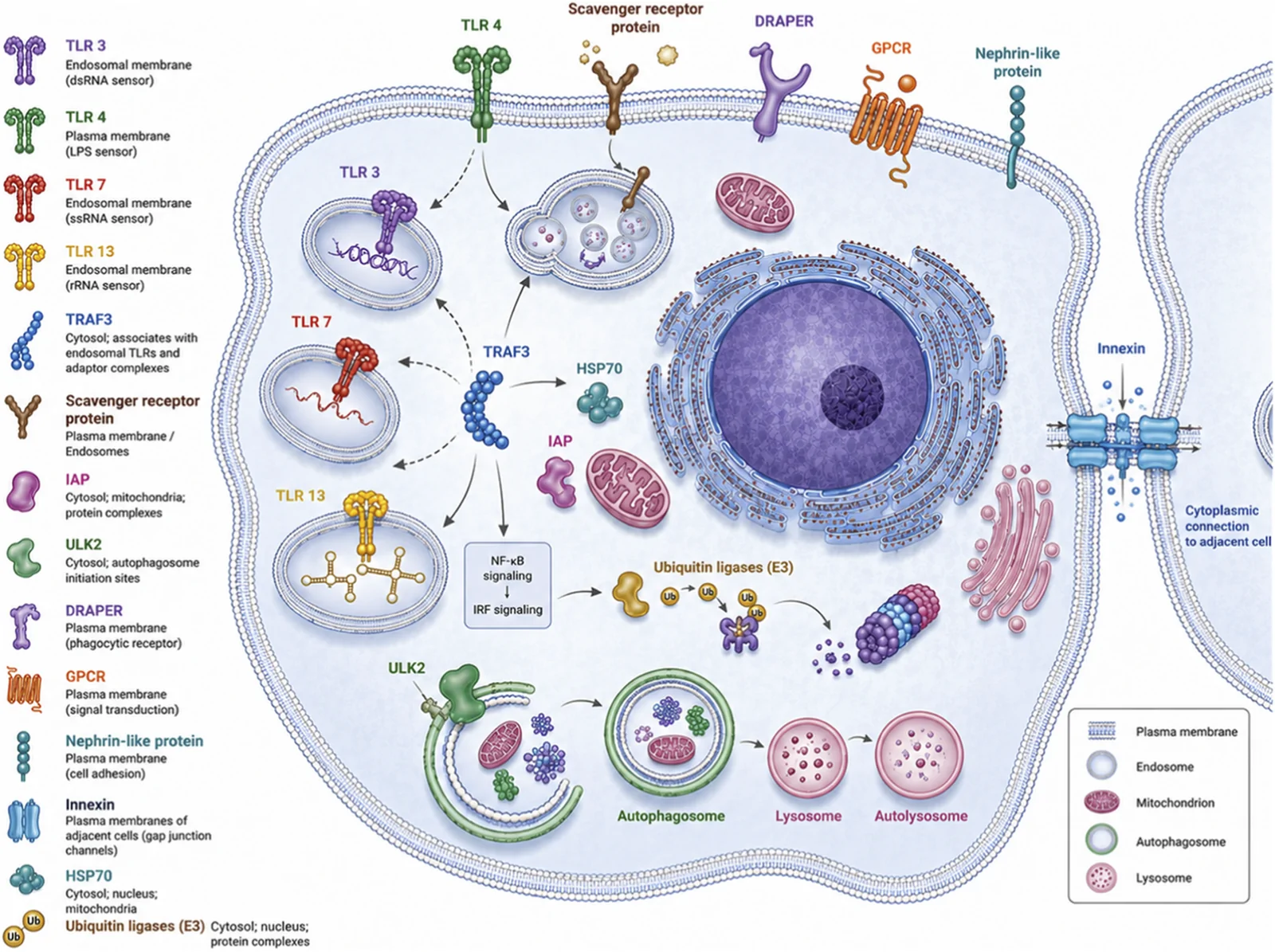
Functional mapping of selected candidate admixed proteins in a representative eukaryotic cell. The proteins illustrated were identified within genomic hotspots of introgression and positioned according to their subcellular localization. Their distribution reveals that several core components of innate immune signalling, together with downstream effectors and regulatory proteins involved in apoptosis, protein homeostasis, autophagy, and intercellular communication, are admixed across a broad range of *Pinna nobilis* individuals. That figure has been generated using AI image-generation tools and subsequently reviewed and manually refined by the authors to ensure biological accuracy.

If some introgressed alleles contribute to increased capacity to cope with MMEs, their effects are unlikely to result in complete resistance, given that nearly all individuals ultimately succumbed to infection, regardless of whether the event was primarily associated with *Haplosporidium pinnae* or potentially with other infectious agents such as bacteria and viruses. One possible explanation is that any phenotypic effect of introgressed alleles is modest and polygenic, influencing susceptibility, disease progression, or the probability of survival rather than providing complete protection. Moreover, their effects are likely to depend on pathogen load (Sauvage et al., 2010), infection severity (Carella et al., 2023a), and the cumulative contribution of multiple introgressed variants carried by each individual (Sauvage et al., 2010; Gutierrez et al., 2018). Under the exceptionally high infection pressure characterizing recent MMEs, even individuals carrying potentially beneficial introgressed alleles may have experienced mortality. These consideration highlights the need for phenotype-based studies to determine whether the candidate introgressed regions identified here are associated with variation in disease susceptibility, progression, or survival.

Interestingly, the findings presented here seem consistent with those recently reported by Carella et al. regarding the susceptibility and immunocompetence of *P. nobilis* to viral infection, particularly by *P. nobilis* picornavirus (PnPV) (Carella et al., 2023b; Carella et al., 2026; Carella et al., 2024). Their studies demonstrated that PnPV infects haemocytes, the principal immune cells of *P. nobilis*, and is associated with profound ultrastructural alterations affecting intracellular membrane organization, vesicular trafficking, mitochondrial integrity, and cytoskeletal architecture, ultimately impairing haemocyte function (Carella et al., 2024). Furthermore, Carella et al. showed that haemocytes from different *P. nobilis* populations differ in their phagocytic capacity and ability to process viral particles, suggesting population-specific variation in immune competence (Carella et al., 2026). Together, these observations that support the hypothesis that PnPV contributes to immunosuppression and may increase host susceptibility to opportunistic pathogens (Künili et al., 2021; Scarpa et al., 2020; Carella et al., 2023a), provide a relevant mechanistic context for the admixture patterns observed here, although the specific pathogens and mechanisms underlying any potential advantage of admixture remain uncertain.

Similarly, although *P. nobilis* can exceptionally survive infection by the apicomplexan parasite *H*. *pinnae*, the genetic basis of this tolerance remains poorly understood. Notably, several admixed genes located within candidate introgressed regions identified in the present study have previously been implicated in host response to other apicomplexan parasites, providing a rationale for investigating whether these regions may also contribute to variation in responses of *P. nobilis* to H. pinnae infection (Coers and Haldar, 2015; Martín-Hernández et al., 2017; Mukhopadhyay et al., 2020; Sambade et al., 2025; Mello-Vieira and Dikic, 2026). However, these functional parallels do not demonstrate that the introgressed regions confer tolerance or resistance to MMEs. Yet, they identify promising candidate genes and pathways that warrant further functional studies to determine whether these genes contribute to resistance or tolerance to *H. pinnae*.

In our dataset, no individuals exhibiting intermediate levels of admixture were detected, suggesting that recent hybridization or subsequent backcrossing is uncommon, although the absence of such individuals could also reflect sampling limitations or reduced hybrid viability. Whether the scarcity of intermediate hybrids results from intrinsic barriers to gene flow or ecological and demographic factors remains to be determined. Further investigations would therefore be valuable to assess whether hybridization rates could increase under conditions favouring sustained contact between the two species, such as the establishment of a stable hybrid zone. This question is particularly relevant given the reported expansion of *P. rudis* populations (Fassio et al., 2025; Kersting and Ballesteros, 2021; Gvozdenović et al., 2019; Oprandi et al., 2024) and ongoing conservation efforts aimed at supporting and potentially increasing *P. nobilis* populations.

### 4.5 Conservation perspectives and future directions

Thau retains substantial genetic diversity at both mitochondrial and nuclear levels, and its admixture profile encompasses variation also detected in Diane lagoon and the studied Open-sea populations, highlighting its potential value as a reservoir of genetic diversity. Given the severe depletion of many Open-sea populations, the Thau lagoon population could potentially contribute to restoring gene flow with depleted open-sea populations. Such connectivity could be re-established naturally through larval dispersal and existing hydrodynamic connections, although the extent and timescale of natural recolonization remain uncertain. Alternatively, assisted gene flow through the translocation of a limited number of individuals from the large Thau population to depleted open-sea populations could be considered to accelerate genetic exchange while minimizing the demographic impact on the source population. Such interventions would nevertheless require careful evaluation of disease risk, recipient population characteristics, and the potential consequences for local genetic composition and adaptation.

Future studies combining genomic, demographic, ecological, and epidemiological data will be important for assessing whether and where assisted gene flow could contribute to the recovery of depleted *P. nobilis* populations. In particular, future work should link genomic variation to infection phenotypes by comparing survivors, highly susceptible individuals, and individuals showing delayed disease progression, while integrating pathogen load, infection progression, and survival data with selection scans and admixture analyses. Such genotype–phenotype data are currently unavailable but are essential to determine whether introgressed or selected genomic regions are associated with variation in disease response and whether they could ultimately inform conservation planning.

## Data Availability

The original contributions presented in the study are publicly available. The raw sequencing reads can be found in the NCBI Sequence Read Archive under BioProject accession numbers PRJNA1527659.

## References

[B1] Aglieri G. Papetti C. Zane L. Milisenda G. Boero F. Piraino S. (2014). First evidence of inbreeding, relatedness and chaotic genetic patchiness in the holoplanktonic jellyfish Pelagia noctiluca (Scyphozoa, Cnidaria). PLoS One 9, e99647. 10.1371/journal.pone.0099647 24977703 PMC4076186

[B2] Akey J. M. Eberle M. A. Rieder M. J. Carlson C. S. Shriver M. D. Nickerson D. A. (2004). Population history and natural selection shape patterns of genetic variation in 132 genes. PLoS Biol. 2, e286. 10.1371/journal.pbio.0020286 15361935 PMC515367

[B3] Alexander D. H. Novembre J. Lange K. (2009). Fast model-based estimation of ancestry in unrelated individuals. Genome Res. 19, 1655–1664. 10.1101/gr.094052.109 19648217 PMC2752134

[B4] Allendorf F. Hössjer O. Ryman N. (2024). What does effective population size tell us about loss of allelic variation? Evol. Appl. 17, e13733. 10.1111/eva.13733 38911263 PMC11192967

[B5] Amos W. Balmford A. (2001). When does conservation genetics matter? Heredity 87, 257–265. 10.1046/j.1365-2540.2001.00940.x 11737272

[B6] Andree K. B. Carrasco N. Carella F. Furones D. Prado P. (2020). Vibrio mediterranei, a potential emerging pathogen of Marine Fauna: investigation of pathogenicity using a bacterial challenge in Pinna nobilis and development of a species-specific PCR. J. Appl. Microbiol. 130 (2), 617–631. 10.1111/jam.14756 32592599

[B7] Audouin J. (1962). Hydrologie de l’étang de Thau. Rev. Trav. Inst. Pêch. Marit. 26, 5–104.

[B8] Azzena I. Locci C. Pascale N. Deplano I. Senigaglia R. Batistini E. (2025). Conservation hints for Pinna nobilis from a century-old genetic time capsule. Sci. Rep. 15, 37660. 10.1038/s41598-025-21574-6 41152429 PMC12569023

[B9] Azzena I. Casu M. Locci C. Deplano I. Pascale N. Senigaglia R. (2026). Between ocean and sea: population genetics and evolutionary history of Pinna rudis. BMC Ecol. Evol. 10.1186/s12862-026-02549-1 42458243

[B10] Bouckaert R. Heled J. Kühnert D. Vaughan T. Wu C. H. Xie D. (2014). BEAST 2: a software platform for Bayesian evolutionary analysis. PLoS Comput. Biol. 10, e1003537. 10.1371/journal.pcbi.1003537 24722319 PMC3985171

[B11] Breton S. Burger G. Stewart D. T. Blier P. U. (2006). Comparative analysis of gender-associated complete mitochondrial genomes in marine mussels (Mytilus spp.). Genetics 172, 1107–1119. 10.1534/genetics.105.047159 16322521 PMC1456209

[B12] Browning B. L. Tian X. Zhou Y. Browning S. R. (2021). Fast two-stage phasing of large-scale sequence data. Am. J. Hum. Genet. 108, 1880–1890. 10.1016/j.ajhg.2021.08.005 PMC8551421 34478634

[B13] Burton R. S. Pereira R. J. Barreto F. S. (2013). Cytonuclear genomic interactions and hybrid breakdown. Annu. Rev. Ecol. Evol. Syst. 44, 281–302. 10.1146/annurev-ecolsys-110512-135758

[B14] Cabanellas-Reboredo M. Vázquez-Luis M. Mourre B. Álvarez E. Deudero S. Amores Á. (2019). Tracking a mass mortality outbreak of pen shell Pinna nobilis populations: a collaborative effort of scientists and citizens. Sci. Rep. 9, 13355. 10.1038/s41598-019-49808-4 31527825 PMC6746856

[B15] Capt C. Bouvet K. Guerra D. Robicheau B. M. Stewart D. T. Pante E. (2020). Unorthodox features in two venerid bivalves with doubly uniparental inheritance of mitochondria. Sci. Rep. 10, 1087. 10.1038/s41598-020-57975-y 31974502 PMC6978325

[B16] Carella F. Palić D. Šarić T. Župan I. Gorgoglione B. Prado P. (2023a). Multipathogen infections and multifactorial pathogenesis involved in noble pen shell (Pinna nobilis) mass mortality events: background and current pathologic approaches. Vet. Pathol. 60, 560–577. 10.1177/03009858231186737 37458195

[B17] Carella F. Prado P. De Vico G. Palić D. Villari G. García-March J. R. (2023b). A widespread picornavirus affects the hemocytes of the noble pen shell (Pinna nobilis), leading to its immunosuppression. Front. Vet. Sci. 10, 1273521. 10.3389/fvets.2023.1273521 PMC10758234 38164394

[B18] Carella F. Prado P. García-March J. R. Tena-Medialdea J. Melendreras E. C. Porcellini A. (2024). Measuring immunocompetence in the natural population and captive individuals of noble pen shell Pinna nobilis affected by Pinna nobilis Picornavirus (PnPV). Fish. Shellfish Immunol. 151, 109664. 10.1016/j.fsi.2024.109664 38844186

[B19] Carella F. Feola A. Romano G. Aceto S. Garcia-March J. R. Tena-Medialdea J. (2026). Interplay between immune cell damage and depletion, ecological factors and *Pinna nobilis* picornavirus (PnPV) infection in the Venice Lagoon (Italy). Preprint. 10.21203/rs.3.rs-9854708/v1

[B20] Catanese G. Vázquez-Luis M. Giacobbe S. García-March J. R. Zotou M. Patricia P. (2024). Internal transcribed spacer as effective molecular marker for the detection of natural hybridization between the bivalves Pinna nobilis and Pinna rudis. Ecol. Evol. 14, e70227. 10.1002/ece3.70227 39193167 PMC11347391

[B21] Catanese G. Coupé S. Bunet R. (2022). Mitogenome sequence comparison in the endangered congeneric Pinna nobilis and Pinna rudis bivalves. Mol. Biol. Rep. 3627–3635. 10.1007/s11033-022-07202-w 35113303

[B22] Coers J. Haldar A. K. (2015). Ubiquitination of pathogen-containing vacuoles promotes host defense to *Chlamydia trachomatis* and Toxoplasma gondii. Commun. Integr. Biol. 8, e1115163. 10.1080/19420889.2015.1115163 27066178 PMC4802790

[B23] Cosentino A. Giacobbe S. (2008). Aspects of epizoobiontic mollusc assemblages on Pinna shells. II. Does the Mediterranean *P. nobilis* represent an isle of biodiversity? Cah. Biol. Mar. 49, 161.

[B24] Coupé S. Giantsis I. A. Vázquez Luis M. Scarpa F. Foulquié M. Prévot J. (2023). The characterization of toll-like receptor repertoire in *Pinna nobilis* after mass mortality events suggests adaptive introgression. Ecol. Evol. 13, e10383. 10.1002/ece3.10383 37546570 PMC10401143

[B25] Coupé S. Foulquié M. Luis M. V. Perez E. A. Prévot J. Vicente N. (2026). Comparative genomics of Pinna rudis and Pinna nobilis reveals conserved and divergent features of the bivalve defensome. Ecol. Evol. 16, e73267. 10.1002/ece3.73267 42016959 PMC13093298

[B26] Couvray S. Coupé S. (2018). Three-year monitoring of genetic diversity reveals a micro-connectivity pattern and local recruitment in the broadcast marine species *Paracentrotus lividus* . Heredity 120, 110–124. 10.1038/s41437-017-0013-6 29180717 PMC5837108

[B27] Danecek P. Auton A. Abecasis G. Albers C. A. Banks E. DePristo M. A. (2011). The variant call format and VCF tools. Bioinformatics 27, 2156–2158. 10.1093/bioinformatics/btr330 21653522 PMC3137218

[B28] Danecek P. Bonfield J. K. Liddle J. Marshall J. Ohan V. Pollard M. O. (2021). Twelve years of SAMtools and BCFtools. Gigascience 10, giab008. 10.1093/gigascience/giab008 PMC7931819 33590861

[B29] Danovaro R. Pusceddu A. (2007). Biodiversity and ecosystem functioning in coastal lagoons: does microbial diversity play any role? Estuar. Coast. Shelf Sci. 75, 4–12. 10.1016/j.ecss.2007.02.030

[B30] Derolez V. Malet N. Fiandrino A. Lagarde F. Richard M. Ouisse V. (2020). Fifty years of ecological changes: regime shifts and drivers in a coastal Mediterranean lagoon during oligotrophication. Sci. Total Environ. 732, 139292. 10.1016/j.scitotenv.2020.139292 32438187

[B31] Durand E. Y. Patterson N. Reich D. Slatkin M. (2011). Testing for ancient admixture between closely related populations. Mol. Biol. Evol. 28, 2239–2252. 10.1093/molbev/msr048 21325092 PMC3144383

[B32] Excoffier L. Ray N. (2008). Surfing during population expansions promotes genetic revolutions and structuration. Trends Ecol. Evol. 23, 347–351. 10.1016/j.tree.2008.04.004 18502536

[B33] Fassio G. Nocella E. Di Ganci R. Cardone S. Farina F. Mancini G. (2025). Dynamics and population genetics of Pinna rudis in the Tyrrhenian Sea: exploring the potential link to the local extinction of Pinna nobilis. Mar. Environ. Res. 213, 107626. 10.1016/j.marenvres.2025.107626 41110442

[B34] Fiandrino A. Giraud A. Robin S. Pinatel C. (2012). Validation d’une méthode d’estimation des volumes d’eau échangés entre la mer et les lagunes et définition d’indicateurs hydrodynamiques associés.

[B35] Figueras A. Moreira R. Sendra M. Novoa B. (2019). Genomics and immunity of the Mediterranean mussel *Mytilus galloprovincialis* in a changing environment. Fish. Shellfish Immunol. 90, 440–445. 10.1016/j.fsi.2019.04.064 31048040

[B36] Fitzpatrick S. W. Bradburd G. S. Kremer C. T. Salerno P. E. Angeloni L. M. Funk W. C. (2020). Genomic and fitness consequences of genetic rescue in wild populations. Curr. Biol. 30, 517–522. 10.1016/j.cub.2019.11.062 31902732

[B37] Foulquié M. Coupé S. Vicente N. Bunet R. (2024). Population status of the critically endangered fan mussel Pinna nobilis in thau Lagoon (France). Endanger. Species Res. 55, 21–36. 10.3354/esr01357

[B38] Foulquié M. Bailet A. Hatret Y. Vicente N. Coupé S. Bunet R. (2026). Surviving the collapse: unprecedented densities of the critically endangered fan mussel Pinna nobilis in a Mediterranean coastal lagoon. Sci. Rep. 16, 18390. 10.1038/s41598-026-48614-z PMC13265810 42009903

[B39] Gignoux-Wolfsohn S. Ruiz M. G. Barron D. P. Ruiz G. Lohan K. (2024). Bivalve microbiomes are shaped by host species, size, parasite infection, and environment. PeerJ 12, e18082. 10.7717/peerj.18082 PMC11468899 39399422

[B40] Goudet J. (2005). Hierfstat, a package for R to compute and test hierarchical F-statistics. Mol. Ecol. Notes 5, 184–186. 10.1111/j.1471-8286.2004.00828.x

[B41] Gravel S. (2012). Population genetics models of local ancestry. Genetics 191, 607–619. 10.1534/genetics.112.139808 22491189 PMC3374321

[B42] Green T. J. Speck P. (2018). Antiviral defense and innate immune memory in the oyster. Viruses 10, 133. 10.3390/v10030133 29547519 PMC5869526

[B43] Guelorget O. Perthuisot J. Lamy N. Lefebvre A. (1994). Structure et organisation de l’étang de Thau d’après la faune benthique (macrofaune, méiofaune). Relations avec le confinement. Oceanol. Acta 17, 105–114.

[B44] Guo X. Ford S. E. (2016). Infectious diseases of marine molluscs and host responses as revealed by genomic tools. Philos. Trans. R. Soc. B Biol. Sci. 371, 20150206. 10.1098/rstb.2015.0206 26880838 PMC4760136

[B45] Gutierrez A. P. Bean T. P. Hooper C. Stenton C. A. Sanders M. B. Paley R. K. (2018). A genome-wide association study for host resistance to ostreid herpesvirus in Pacific oysters (*Crassostrea gigas*). G3 Genes Genomes Genet. 8, 1273–1280. 10.1534/g3.118.200113 29472307 PMC5873916

[B46] Gvozdenović S. Macic V. Pesic V. Nikolic M. Peras I. Mandic M. (2019). Review on *Pinna rudis* (Linnaeus, 1758)(bivalvia: pinnidae) presence in the Mediterranean. Poljopr. Sumar. 65, 115–126. 10.17707/agricultforest.65.4.10

[B47] Hage A. Rajsbaum R. (2019). To TRIM or not to TRIM: the balance of host–virus interactions mediated by the ubiquitin system. J. Gen. Virol. 100, 1641–1662. 10.1099/jgv.0.001341 31661051 PMC7011758

[B48] Hernandis Caballero S. (2021). Avances en la ecología y biología para la conservación de la especie críticamente amenazada Pinna nobilis (Linnaeus, 1758), endemismo mediterráneo. Valencia, Spain: University of Valencia. Doctoral Thesis.

[B49] Hill G. E. (2015). Mitonuclear ecology. Mol. Biol. Evol. 32, 1917–1927. 10.1093/molbev/msv104 25931514 PMC4833085

[B50] Jombart T. Ahmed I. (2011). Adegenet 1.3-1: new tools for the analysis of genome-wide SNP data. Bioinformatics 27, 3070–3071. 10.1093/bioinformatics/btr521 21926124 PMC3198581

[B51] Karadurmuş U. Akkuş M. Sarı M. (2025). Distribution of Fan mussel (*Pinna nobilis*) population in the Sea of Marmara. Aquat. Conserv. Mar. Freshw. Ecosyst. 35, e70173. 10.1002/aqc.70173

[B52] Kersting D. K. Ballesteros E. (2021). Is the local extinction of *Pinna nobilis* facilitating *Pinna rudis* recruitment?. Mediterranean Marine Science 22, 623. 10.12681/mms.27809

[B53] Kersting D. K. Vázquez-Luis M. Mourre B. Belkhamssa F. Z. Álvarez E. Bakran-Petricioli T. (2020). Recruitment disruption and the role of unaffected populations for potential recovery after the Pinna nobilis mass mortality event. Front. Mar. Sci. 7, 594378. 10.3389/fmars.2020.594378

[B54] Klopfenstein D. V. Zhang L. Pedersen B. S. Ramírez F. Warwick Vesztrocy A. Naldi A. (2018). GOATOOLS: a python library for gene ontology analyses. Sci. Rep. 8, 10872. 10.1038/s41598-018-28948-z 30022098 PMC6052049

[B55] Künili İ. E. Ertürk Gürkan S. Aksu A. Turgay E. Çakir F. Gürkan M. (2021). Mass mortality in endangered fan mussels Pinna nobilis (Linnaeus 1758) caused by co-infection of Haplosporidium pinnae and multiple Vibrio infection in Çanakkale Strait, Turkey. Biomarkers 26, 1–12. 10.1080/1354750x.2021.1910344 33899623

[B56] Lattos A. Coupé S. Papadopoulos D. K. Giantsis I. A. (2025). Transcriptional regulation of toll-like receptors in Pinna nobilis: insights into immune response under varying pathogen loads and effect of individual variation. Gene Rep. 42, 102415. 10.1016/j.genrep.2025.102415

[B57] Lawson D. J. Van Dorp L. Falush D. (2018). A tutorial on how not to over-interpret STRUCTURE and ADMIXTURE bar plots. Nat. Commun. 9, 3258. 10.1038/s41467-018-05257-7 30108219 PMC6092366

[B58] Lazure P. (1992). Etude de la dynamique de l’étang de Thau par modèle numérique tridimensionnel. Vie MilieuLife Environ., 137–145.

[B59] Leigh J. W. Bryant D. Nakagawa S. (2015). POPART: full-feature software for haplotype network construction. Methods Ecol. Evol. 6, 1110. 10.1111/2041-210X.12410

[B60] Li H. Durbin R. (2009). Fast and accurate short read alignment with Burrows–wheeler transform. Bioinformatics 25, 1754–1760. 10.1093/bioinformatics/btp324 19451168 PMC2705234

[B61] Li A. Dai H. Guo X. Zhang Z. Zhang K. Wang C. (2021). Genome of the estuarine oyster provides insights into climate impact and adaptive plasticity. Commun. Biol. 4, 1287. 10.1038/s42003-021-02823-6 PMC8590024 34773106

[B62] Liang M. Nielsen R. (2014). The lengths of admixture tracts. Genetics 197, 953–967. 10.1534/genetics.114.162362 24770332 PMC4096373

[B63] Lin J. Guo M. Li C. (2026). An overview of post-translational modifications in aquatic animal immunity and pathogen evasion. Rev. Aquac. 18, e70182. 10.1111/raq.70182

[B64] Lopes E. P. Santos S. Xavier R. Santos J. L. Cabezas M. P. Sequeira F. (2025). Genetic analysis of Pinna rudis L 1758 (Mollusca, Bivalvia, Pinnidae) in the Northwest Cabo Verde Islands (Central-East Atlantic). PeerJ 13, e18328. 10.7717/peerj.18328 PMC11724654 39802188

[B65] Luu K. Bazin E. Blum M. G. (2017). Pcadapt: an R package to perform genome scans for selection based on principal component analysis. Mol. Ecol. Resour. 17, 67–77. 10.1111/1755-0998.12592 27601374

[B66] Maples B. K. Gravel S. Kenny E. E. Bustamante C. D. (2013). RFMix: a discriminative modeling approach for rapid and robust local-ancestry inference. Am. J. Hum. Genet. 93, 278–288. 10.1016/j.ajhg.2013.06.020 23910464 PMC3738819

[B67] Marcais G. Kingsford C. (2012). Jellyfish: a fast k-mer counter. Tutorialis E Manuais 1, 1–8.

[B68] Maresca F. Álvarez E. Zafra L. Hendriks I. E. Catanese G. González R. (2025). Chasing Pinna nobilis survivors: current status in Spanish open Coastal waters. Animals 15, 3075. 10.3390/ani15213075 PMC12607348 41227405

[B69] Martin S. H. Jiggins C. D. (2017). Interpreting the genomic landscape of introgression. Curr. Opin. Genet. Dev. 47, 69–74. 10.1016/j.gde.2017.08.007 28923541

[B70] Martín-Hernández R. Higes M. Sagastume S. Juarranz Á. Dias-Almeida J. Budge G. E. (2017). Microsporidia infection impacts the host cell’s cycle and reduces host cell apoptosis. PloS One 12, e0170183. 10.1371/journal.pone.0170183 28152065 PMC5289437

[B71] Martínez-Martínez P. Cortés-Melendreras E. Fernández-Torquemada Y. Barberá C. del-Pilar-Ruso Y. Izquierdo-Muñoz A. (2024). Assessing habitat microscale conditions underlying with mortality and survival of Pinna nobilis in the Mar Menor coastal lagoon (SE Spain). J. Sea Res. 200, 102518. 10.1016/j.seares.2024.102518

[B72] Mello-Vieira J. Dikic I. (2026). Ubiquitination and autophagy in host–pathogen interactions: from immune surveillance to therapeutic targeting. Nat. Rev. Immunol. 335–349. 10.1038/s41577-025-01239-1 41491841

[B73] Mihaljević Ž. Pavlinec Ž. Zupičić I. G. Oraić D. Popijač A. Pećar O. (2021). Noble pen shell (Pinna nobilis) mortalities along the Eastern adriatic Coast with a study of the spreading velocity. J. Mar. Sci. Eng. 9, 764. 10.3390/jmse9070764

[B74] Millet B. (1989). Fonctionnement hydrodynamique du bassin de Thau. Validation écologique d’un modèle numérique de circulation (programme Ecothau). Oceanol. Acta 12, 37–46.

[B75] Mukherjee R. Dikic I. (2022). Regulation of host-pathogen interactions via the ubiquitin system. Annu. Rev. Microbiol. 76, 211–233. 10.1146/annurev-micro-041020-025803 36075095

[B76] Mukhopadhyay D. Sangaré L. O. Braun L. Hakimi M. Saeij J. P. (2020). Toxoplasma GRA15 limits parasite growth in IFNγ-activated fibroblasts through TRAF ubiquitin ligases. EMBO J. 39, EMBJ2019103758. 10.15252/embj.2019103758 32293748 PMC7232000

[B77] Nebot-Colomer E. Peyran C. Deudero S. Boissin E. Vázquez-Luis M. Planes S. (2025). The mass mortality of Pinna nobilis throughout the Mediterranean Sea has not yet affected the genetic diversity of the species in one of the last genetic reservoirs. Estuar. Coast. Shelf Sci. 317, 109202. 10.1016/j.ecss.2025.109202

[B78] Nielsen R. (2005). Molecular signatures of natural selection. Annu. Rev. Genet. 39, 197–218. 10.1146/annurev.genet.39.073003.112420 16285858

[B79] Oprandi A. Aicardi S. Azzola A. Benelli F. Bertolino M. Bianchi C. N. (2024). A tale of two sisters: the southerner Pinna rudis is getting North after the regional extinction of the congeneric *P. nobilis* (Mollusca: Bivalvia). Diversity 16, 120. 10.3390/d16020120

[B80] Pensa D. Fianchini A. Grosso L. Ventura D. Cataudella S. Scardi M. (2022). Population status, distribution and trophic implications of Pinna nobilis along the South-eastern Italian coast. Npj Biodivers. 1, 3. 10.1038/s44185-022-00002-2 PMC11290603 39242780

[B81] Pérez-Ruzafa A. Marcos C. Pérez-Ruzafa I. M. Pérez-Marcos M. (2011). Coastal lagoons:“transitional ecosystems” between transitional and coastal waters. J. Coast. Conserv. 15, 369–392. 10.1007/s11852-010-0095-2

[B82] Peyran C. Boissin E. Morage T. Nebot-Colomer E. Iwankow G. Planes S. (2021). Genetic homogeneity of the critically endangered fan mussel, Pinna nobilis, throughout lagoons of the Gulf of Lion (North-Western Mediterranean Sea). Sci. Rep. 11, 1–12. 10.1038/s41598-021-87493-4 PMC8032772 33833376

[B83] Peyran C. Morage T. Nebot-Colomer E. Iwankow G. Planes S. (2022). Unexpected residual habitats raise hope for the survival of the fan mussel Pinna nobilis along the Occitan coast (Northwest Mediterranean Sea). Endanger. Species Res. 48, 123–137. 10.3354/esr01191

[B84] Purcell S. Neale B. Todd-Brown K. Thomas L. Ferreira M. A. R. Bender D. (2007). PLINK: a tool set for whole-genome association and population-based linkage analyses. Am. J. Hum. Genet. 81, 559–575. 10.1086/519795 17701901 PMC1950838

[B85] Quero G. M. Perini L. Pesole G. Manzari C. Lionetti C. Bastianini M. (2017). Seasonal rather than spatial variability drives planktonic and benthic bacterial diversity in a microtidal lagoon and the adjacent open sea. Mol. Ecol. 26, 5961–5973. 10.1111/mec.14363 28926207

[B86] Quinlan A. R. Hall I. M. (2010). BEDTools: a flexible suite of utilities for comparing genomic features. Bioinformatics 26, 841–842. 10.1093/bioinformatics/btq033 20110278 PMC2832824

[B87] Rabaoui L. Tlig-Zouari S. Cosentino A. Hassine O. K. B. (2009). Associated fauna of the fan shell Pinna nobilis (Mollusca: Bivalvia) in the northern and eastern Tunisian coasts. Sci. Mar. 73, 129–141. 10.3989/scimar.2009.73n1129

[B88] Racimo F. Sankararaman S. Nielsen R. Huerta-Sánchez E. (2015). Evidence for archaic adaptive introgression in humans. Nat. Rev. Genet. 16, 359–371. 10.1038/nrg3936 25963373 PMC4478293

[B89] Ranallo-Benavidez T. R. Jaron K. S. Schatz M. C. (2020). GenomeScope 2.0 and Smudgeplot for reference-free profiling of polyploid genomes. Nat. Commun. 11, 1432. 10.1038/s41467-020-14998-3 32188846 PMC7080791

[B90] Rouanet E. Trigos S. Vicente N. (2015). From youth to death of old age: the 50-year story of a Pinna nobilis fan mussel population at Port-Cros Island (Port-Cros National Park, Provence, Mediterranean Sea). Sci. Rep. Port-Cros Natl. Park 29, 209–222.

[B91] Rozas J. Ferrer-Mata A. Sánchez-DelBarrio J. C. Guirao-Rico S. Librado P. Ramos-Onsins S. E. (2017). DnaSP 6: DNA sequence polymorphism analysis of large data sets. Mol. Biol. Evol. 34, 3299–3302. 10.1093/molbev/msx248 29029172

[B92] Saco A. Novoa B. Greco S. Gerdol M. Figueras A. (2023). Bivalves present the largest and most diversified repertoire of toll-like receptors in the animal kingdom, suggesting broad-spectrum pathogen recognition in marine waters. Mol. Biol. Evol. 40, msad133. 10.1093/molbev/msad133 PMC10279657 37279919

[B93] Sambade I. M. Estêvão J. Pampín M. Cruz A. Guévélou E. Blanco A. (2025). Signatures of selection for resistance/tolerance to Perkinsus olseni in Grooved Carpet Shell Clam (Ruditapes decussatus) using a population genomics approach. Evol. Appl. 18, e70106. 10.1111/eva.70106 40365167 PMC12070250

[B94] Sanna D. Cossu P. Dedola G. L. Scarpa F. Maltagliati F. Castelli A. (2013). Mitochondrial DNA reveals genetic structuring of Pinna nobilis across the Mediterranean Sea. PLoS One 8, e67372. 10.1371/journal.pone.0067372 23840684 PMC3696058

[B95] Sanna D. Azzena I. Locci C. Ankon P. Kružić P. Manfrin C. (2023). Reconstructing the evolutionary history of Pinna nobilis: new genetic signals from the past of a species on the brink of extinction. Animals 14, 114. 10.3390/ani14010114 38200845 PMC10778441

[B96] Sarafidou G. Tsaparis D. Issaris Y. Chatzigeorgiou G. Grigoriou P. Chatzinikolaou E. (2023). Insights on Pinna nobilis population genetic structure in the Aegean and Ionian Sea. PeerJ 11, e16491. 10.7717/peerj.16491 38047017 PMC10693241

[B97] Sauvage C. Boudry P. de Koning D. J. Haley C. S. Heurtebise S. Lapègue S. (2010). QTL for resistance to summer mortality and OsHV-1 load in the Pacific oyster (*Crassostrea gigas*). Anim. Genet. 41, 390–399. 10.1111/j.1365-2052.2009.02018.x 20096029

[B98] Scarpa F. Sanna D. Azzena I. Mugetti D. Cerruti F. Hosseini S. (2020). Multiple non-species-specific pathogens possibly triggered the mass mortality in Pinna nobilis. Life 10, 238. 10.3390/life10100238 33066230 PMC7650684

[B99] Song K. Wen S. Zhang G. (2019). Adaptive evolution patterns in the pacific oyster *Crassostrea gigas* . Mar. Biotechnol. 21, 614–622. 10.1007/s10126-019-09906-w 31203476

[B100] Takeuchi T. Suzuki Y. Watabe S. Nagai K. Masaoka T. Fujie M. (2022). A high-quality, haplotype-phased genome reconstruction reveals unexpected haplotype diversity in a pearl oyster. DNA Res. 29, dsac035. 10.1093/dnares/dsac035 36351462 PMC9646362

[B101] Thorvaldsdóttir H. Robinson J. T. Mesirov J. P. (2013). Integrative genomics viewer (IGV): high-performance genomics data visualization and exploration. Brief. Bioinform. 14, 178–192. 10.1093/bib/bbs017 22517427 PMC3603213

[B102] Trigos S. García-March J. Vicente N. Tena J. Torres J. (2014). Utilization of muddy detritus as organic matter source by the fan mussel Pinna nobilis. Mediterr. Mar. Sci. 667–674. 10.12681/mms.836

[B103] Trombetta T. Bouget F.-Y. Félix C. Mostajir B. Vidussi F. (2022). Microbial diversity in a north Western Mediterranean Sea shallow coastal lagoon under contrasting water temperature conditions. Front. Mar. Sci. 9, 858744. 10.3389/fmars.2022.858744

[B104] Vázquez-Luis M. Nebot-Colomer E. Deudero S. Planes S. Boissin E. (2021). Natural hybridization between pen shell species: Pinna rudis and the critically endangered Pinna nobilis may explain parasite resistance in *P. nobilis* . Mol. Biol. Rep. 48, 997–1004. 10.1007/s11033-020-06063-5 33394229

[B105] Vendrami D. L. Telesca L. Weigand H. Weiss M. Fawcett K. Lehman K. (2017). RAD sequencing resolves fine-scale population structure in a benthic invertebrate: implications for understanding phenotypic plasticity. R. Soc. Open Sci. 4, 160548. 10.1098/rsos.160548 28386419 PMC5367306

[B106] Vetri A. Basso A. D’Onofrio C. Pretto T. Turolla E. Marcer F. (2026). First report of Haplosporidium edule infection in the olive-green cockle (Cerastoderma glaucum) from the Northern adriatic Sea: expanding host range and geographic distribution. Pathogens 15 (4), 415. 10.3390/pathogens15040415 PMC13119199 42075742

[B107] Wakeley J. (2009). Coalescent theory: an introduction. Greenwood Village, CO: Roberts & Company Publishers.

[B108] Wendling C. C. Wegner K. M. (2015). Adaptation to enemy shifts: rapid resistance evolution to local Vibrio spp. in invasive Pacific oysters. Proc. R. Soc. B Biol. Sci. 282, 20142244. 10.1098/rspb.2014.2244 25716784 PMC4375854

[B109] Wesselmann M. González-Wangüemert M. Serrão E. A. Engelen A. H. Renault L. García-March J. R. (2018). Genetic and oceanographic tools reveal high population connectivity and diversity in the endangered pen shell Pinna nobilis. Sci. Rep. 8, 1–11. 10.1038/s41598-018-23004-2 29555926 PMC5859023

[B110] Whiteley A. R. Fitzpatrick S. W. Funk W. C. Tallmon D. A. (2015). Genetic rescue to the rescue. Trends Ecol. Evol. 30, 42–49. 10.1016/j.tree.2014.10.009 25435267

[B111] Wooldridge T. B. Ford S. M. Conwell H. C. Hyde J. Harris K. Shapiro B. (2025). Direct measurement of the mutation rate and its evolutionary consequences in a critically endangered mollusk. Mol. Biol. Evol. 42, msae266. 10.1093/molbev/msae266 39775835 PMC11704959

[B112] Zavodnik D. Hrs-Brenko M. Legac M. (1991). Synopsis on the fan shell Pinna nobilis L. in the eastern Adriatic Sea. Espèces Mar. À Prot. En. Méditerranée, 169–178.

[B113] Zhang G. Fang X. Guo X. Li L. Luo R. Xu F. (2012). The oyster genome reveals stress adaptation and complexity of shell formation. Nature 490, 49–54. 10.1038/nature11413 22992520

[B114] Zimmerman S. J. Aldridge C. L. Oyler-McCance S. J. (2020). An empirical comparison of population genetic analyses using microsatellite and SNP data for a species of conservation concern. BMC Genomics 21, 382. 10.1186/s12864-020-06783-9 32487020 PMC7268520

