# Supplementary material for: Genome-wide diversity and Pinna rudis admixture reveal Thau lagoon as a conservation reservoir for the collapsing fan mussel Pinna nobilis
Source: Front Genet. 2026 Oct 26;17:1970183. doi: 10.3389/fgene.2026.1970183 (PMC13745860; doi:10.3389/fgene.2026.1970183)
Supplement: Supplementary file 1 [file Supplementaryfile1.docx]

**Genome-wide diversity and *Pinna rudis* admixture reveal Thau Lagoon as a conservation reservoir for the collapsing fan mussel *Pinna nobilis***

**Mathieu Foulquié^1,2^, Robert Bunet^2^, Maite Vázquez Luis^3^, Ioannis A. Giantsis^4^, Fabio Scarpa^5^, Daria Sanna^5^, Elvira Alvarez Perez^3^, Marco Casu^6^, Jean-Marc Prévot^7^, Nardo Vicente^2,8^ & Stéphane Coupé^1^**

1. Université de Toulon, Aix Marseille Univ, CNRS, IRD, MIO, Marseille, France.

2. Institut océanographique Paul Ricard, Ile des Embiez, Var, France.

3. Instituto Español de Oceanografía (IEO, CSIC), Centro Oceanográfico de Baleares. Muelle de poniente s/n, 07015. Palma de Mallorca, Spain.

4. Laboratory of Ichthyology and Fisheries, Faculty of Agriculture, Forestry and Natural Environment, Aristotle University of Thessaloniki, 54124 Thessaloniki, Greece.

5. University of Sassari, Department of Biomedical Sciences, 07100 Sassari (Italy).

6. University of Sassari, Department of Innovation, 07026 Olbia (Italy).

7. Département informatique, Université de Toulon, Var, France.

8. Institut Méditerranéen de Biodiversité et Ecologie marine et continentale (IMBE), Aix-Marseille Université, CNRS, IRD, Avignon Université, France

To whom correspondence should be addressed. Email: [mathieufoulquie@gmail.com](mailto:mathieufoulquie@gmail.com); [stephane.coupe@mio.osupytheas.fr](mailto:stephane.coupe@mio.osupytheas.fr)

**Supplementary Figure 1. Overview of nuclear variant processing workflow.**

**Supplementary Figure 2. Haplotype networks of *16S rRNA* (top) and *cox1* (bottom).** Both networks include our newly generated sequences together with all publicly available sequences retrieved from the NCBI database, classified according to sampling date as before or after 2018. For 16S rRNA, all available sequences originated from individuals sampled before 2018.

**Supplementary Figure 3. Mismatch distributions of pairwise nucleotide differences for the three studied populations.** Observed (solid blue line) and expected (dashed red line) mismatch distributions under the growth-decline demographic model for the three populations analysed. The observed distributions exhibit varying degrees of concordance with the theoretical expectation. While some populations display a broadly unimodal distribution consistent with demographic expansion, others show ragged or irregular patterns that may reflect stochastic variation, population structure, or more complex demographic histories.

**Supplementary Figure 4. Demographic fluctuations in effective population size through time assessed with mitochondrial markers.** Effective population size is shown as a function of mutations per site per year, allowing expansion times to be inferred for both the *16S rRNA* and PCG markers using appropriate mutation rates. The continuous line represents the median estimate, and the shaded areas indicate the highest posterior density (HPD) 95% intervals. The most recent time is shown on the left side of the x-axis.
